# Prepubertal Diabetes Stagnates Testicular Development by Skewing Autophagy Homeostasis in Leydig Cells

**DOI:** 10.3390/cells14171376

**Published:** 2025-09-04

**Authors:** Zonghao Tang, Youkun Zheng

**Affiliations:** Basic Medicine Research Innovation Center for Cardiometabolic Diseases, Ministry of Education, Southwest Medical University, Luzhou 646000, China; tangzonghao@163.com

**Keywords:** diabetes, testicular development, Leydig cell, autophagy, inflammation

## Abstract

The maturation of testicular Leydig cells during the prepubertal stage is crucial for establishing male fertility. While diabetes is recognized as a significant detrimental factor affecting male testicular function, its impact specifically during the prepubertal period remains largely unknown. We hypothesized that prepubertal diabetes may impair testicular development by disrupting Leydig cell maturation. Using streptozotocin (STZ) administration, we established a prepubertal diabetic rat model and investigated the effects of diabetes on testicular development 2 and 4 weeks post-STZ treatment. Diabetes significantly hampered testicular development, manifesting as a decreased testicular weight, structural abnormalities, reduced testosterone levels, and increased inflammatory responses. As anticipated, prepubertal diabetes stagnated Leydig cell maturation and increased Leydig cell apoptosis. Mechanistic studies revealed that autophagy is essential for maintaining homeostasis and facilitating differentiation in immature Leydig cells but is significantly inhibited by hyperglycemia. Dysregulation of autophagy impaired the mitochondrial network, triggering inflammatory responses, suppressing steroidogenic capacity, and accumulating reactive oxygen species (ROS). Elevated ROS levels exacerbated the inflammatory response in the Leydig cells in an NLRP3-dependent manner. Inhibition of NLRP3 ameliorated the hyperglycemia-induced inflammation and decline in steroidogenic ability. Collectively, these findings demonstrate that hyperglycemia suppresses autophagy induction and enhances ROS accumulation in Leydig cells. This cascade promotes inflammation and inhibits steroidogenesis, thereby impeding testicular development in prepubertal diabetic rats.

## 1. Introduction

Diabetes is a globally prevalent metabolic disease with an increasing incidence. Substantial evidence demonstrates that diabetes inevitably causes complications affecting multiple organ systems, including the central nervous system, the kidneys, and the vasculature [1,2]. Ubiquitous pathological features of these complications encompass cellular dysfunction, apoptosis, and, notably, inflammation [3]. Diabetic complications arise from the interplay of multiple factors. Hyperglycemia is a primary contributor to cellular stress and dysfunction in diabetes. In males, the progression of diabetes severely disrupts testicular function, ultimately leading to infertility [4]. Reduced testosterone levels are a major driver of diabetes-induced male infertility. Testosterone—essential for spermatogenesis—directly activates downstream kinases in the Sertoli cells, enabling spermatogenic cell proliferation and differentiation [5]. Leydig cells represent the primary, if not sole, source of testicular testosterone, despite comprising only ~2.7% of the testicular volume [6]. In male rats, Leydig cells progressively enhance the testosterone synthesis capacity from postnatal day 21, peaking at puberty’s conclusion [7]. During maturation, the Leydig cell morphology transitions from spindle-shaped to polygonal as the cells migrate toward the central interstitium; this maturation is critical for lifelong male fertility [8]. Diabetes fundamentally disrupts the prepubertal hormonal balance in both sexes [9,10], inducing significant histological damage to the male reproductive system, particularly testicular structure and function [11]. Prepubertal diabetes severely impairs testicular growth in rats [12], with oxidative stress and inflammation—key mechanisms in diabetes complications—mediating testicular impairment [13]. Ample evidence indicates that autophagy mitigates injury due to adverse stimuli in mammalian cells [14]. Nevertheless, whether diabetes exacerbates prepubertal Leydig cell maturation in rats remains unknown.

Autophagy is an evolutionarily conserved self-degradation mechanism in eukaryotes which eliminates redundant cellular components to support cell survival under stressful conditions [15]. However, dysregulated autophagy exerts detrimental effects on cellular functions, contributing to the progression of numerous human diseases, including cancer, Alzheimer’s disease, and diabetes [16]. In the testis, autophagy plays a critical role in regulating Sertoli cell morphology and spermatocyte differentiation, essential processes for establishing male fertility [17]. Notably, steroidogenic cells display elevated autophagic activity compared to that in other cell types under basal conditions [18]. In Leydig cells, autophagy is directly implicated in steroidogenesis, and a reduction in autophagic activity is associated with a diminished steroidogenic capacity [19]. Moreover, during testicular development, the disruption of autophagy in the Leydig cells also impairs sexual behavior in male mice [20], further underscoring the importance of autophagy in Leydig cell function and testicular development. Therefore, elucidating the role of autophagy in testicular pathogenesis in diabetic conditions is imperative.

Based on these findings, we hypothesized that diabetes attenuates the induction of autophagy, leading to impaired metabolic homeostasis and, subsequently, Leydig cell apoptosis and inflammation. To test this hypothesis, a diabetic rat model was established through streptozotocin (STZ) administration. Our results demonstrated that diabetes significantly impaired normal testicular development in male rats. Furthermore, hyperglycemia markedly suppressed autophagy induction, resulting in impaired mitochondrial homeostasis and increased reactive oxygen species (ROS) levels. This aggravated inflammation through the activation of the NLRP3 inflammasome in the Leydig cells. Furthermore, the elevated NLRP3 activity adversely affected both steroidogenesis and Leydig cell maturation.

## 2. Materials and Methods

### 2.1. The Animals and Treatment

The three-week-old male Sprague-Dawley (SD) rats used in the present study were purchased from SPF Biotechnology Co., Ltd. (Beijing, China). The protocol for animal care and use was performed according to the Guide for the Care and Use of Laboratory Animals from the National Institutes of Health and was approved by the Animal Ethical and Welfare Committee of Southwest Medical University. The animals were maintained under a constant temperature of 21–25 °C, a relative humidity of 40–60% and a 12 h light/dark cycle and fed a standard diet and tap water ad libitum. The rats were housed in cages, and each cage contained 4 rats. The diabetic model was created through STZ (80 mg/kg, Sigma, St. Louis, MO, USA) injection (i.p.), whereas the control rats were injected with a vehicle (citrate buffer, pH = 4.5, Macklin, Shanghai, China). One week after the injections, blood glucose levels were measured by applying tail blood to a glucometer. Rats with blood glucose levels > 16.5 mmol/L were considered to be diabetic. All rats were sacrificed two or four weeks after the STZ treatment. The serum was separated and collected for the testosterone analysis. The testes were also harvested immediately after death for Leydig cell isolation or paraformaldehyde (PFA, Macklin, Shanghai, China) fixation.

### 2.2. Blood Glucose Measurements and Serum Testosterone Detection

Blood glucose was measured using a handheld glucometer (Roche, Basel, Switzerland) with appropriate test strips (Roche, Basel, Switzerland). For the serum testosterone analysis, the rats were anaesthetized using pentobarbital (Aladdin, Shanghai, China), and their blood was collected via vena cava puncture. The blood samples were then incubated at room temperature for 20 min, and serum was isolated through centrifugation at 1200× *g* for 10 min. The concentration of testosterone in the serum was determined using an enzyme-linked immunosorbent assay (ELISA) kit (ab108666, Abcam, Cambridge, UK) according to the manufacturer’s manual.

### 2.3. Hematoxylin and Eosin (H&E) and Immunofluorescence

The testes were collected 2 weeks or 4 weeks after the STZ injection, and samples were fixed with 4% PFA in phosphate-buffered saline (PBS, Macklin, Shanghai, China). Then, the samples were dehydrated using gradient alcohol and embedded with paraffin. For H&E staining, 5 μm sections were dewaxed, hydrated, stained with hematoxylin and eosin, and eventually observed under a light microscope. The diameter of the seminiferous tubules was measured according to the method in reference [21]. For immunofluorescence testing, after dewaxing and hydrating the sections, they were placed in a steamer for antigen retrieval for 30 min in 10 mM citric buffer. After that, the sections were incubated in 3% H_2_O_2_ (Macklin, Shanghai, China) for 15 min and then blocked with 5% bovine serum albumin (BSA, Aladdin, Shanghai, China) for 45 min. Subsequently, the sections were incubated at 4 °C overnight with an anti-LC3 rabbit polyclonal antibody (diluted 1:100; ab48394, Abcam, Cambridge, UK) or an anti-cleaved caspase 3 rabbit polyclonal antibody (diluted 1:200; 9664S, Cell Signaling Technology, Boston, MA, USA). After washing, the sections were incubated with the Alexa 594-conjugated secondary antibody (diluted 1:500, Invitrogen, Carlsbad, CA, USA) or the Alexa 488-conjugated secondary antibody (diluted 1:500, Invitrogen, Carlsbad, CA, USA). Finally, slides were mounted with mounting media (Beyotime, Haimen, China), and images were captured using a microscope (AMG Mill Creek, Washington, DC, USA).

### 2.4. Leydig Cell Isolation and In Vitro Culture

Primary rat Leydig cells were collected from the rat testes using Percoll gradient centrifugation [22,23]. Briefly, the testes were removed from immature SD rats (day 21), and decapsulated testes were incubated with 0.1% type I collagenase (C0130, Sigma, St. Louis, MO, USA) for 30 min at 34 °C. After that, crude Leydig cells were filtered using a 70 μm strainer (Sigma, St. Louis, MO, USA) and then collected through centrifugation at 300 g for 10 min. Then, the cells were washed twice in PBS containing 0.1% (*w*/*v*) fetal bovine serum (FBS, Gibco, New York, NY, USA). To obtain purified Leydig cells, the samples were suspended with PBS containing 5% (*w*/*v*) FBS and then loaded on top of a Percoll gradient (20%, 40%, 60%, and 90% Percoll in PBS, *v*/*v*) at 800 g for 30 min at 4 °C. Subsequently, the 60% Percoll (P8370, Solarbio, Beijing, China) fraction was collected and transferred into a 15 mL centrifuge tube. The Percoll buffer was diluted with PBS and centrifuged using a swing-out rotor centrifuge (Eppendorf, Hamburg, Germany) at 500 g for 5 min to pellet the Leydig cells. The pellet was resuspended using Dulbecco’s modified eagle medium (DMEM, Gibco, New York, NY, USA), and purified Leydig cells were eventually collected after configuration at 500 g for 5 min. The purity of the Leydig cells was confirmed by observing the cell morphology under an optical microscope (ZEISS, Oberkochen, Germany). When almost all of the cells were observed to have a consistent morphology, then we proceeded to the subsequent experiments. Cell viability was assessed through Trypan blue dye exclusion. The cells (95% viability) were cultured in DMEM (Gibco, New York, NY, USA ) supplemented with 10% FBS (10270, Gibco, Gibco, New York, NY, USA) and 5.5 mM D-glucose (Aladdin, Shanghai, China) at 37 °C in a humidified atmosphere of 5% CO_2_. For each experiment, 1.5 × 10^6^ cells were plated into a six-well plate to a volume of 2 mL. To study the effect of hyperglycemia on the physiology of the Leydig cells, the cells were cultured for 72 h in glucose-free DMEM supplemented with 5.5 mM, 15 mM, 25 mM, and 50 mM of glucose. For the autophagy study, the cells were treated with and without 3-methyladenine (3-MA) (M9281, Sigma, St. Louis, MO, USA) at a concentration of 2 mM for 24 h. All media were supplied with 100 units/mL penicillin and streptomycin (Beyotime, Haimen, China). Cell viability was assessed using the Cell Counting Kit-8 (CCK-8) (C0037, Beyotime, Haimen, China), following the manufacturer’s instructions. The absorbance was measured at 450 nm using a microplate reader (Eppendorf, Hamburg, Germany) after 2 h of incubation with the reagent. Flow cytometry was applied to assessing apoptosis (Annexin V/PI staining).

### 2.5. Western Blot Analysis

The Leydig cells were lysed using RIPA buffer (P0013B, Beyotime, Haimen, China). The protein samples from the cultured cells were also extracted using RIPA buffer. The protein contents were measured using the BCA kit (23227, Thermo Fisher, Waltham, MA, USA). Protein samples of 40 μg were loaded for sodium dodecyl sulfate–polyacrylamide gel electrophoresis (SDS-PAGE) and then electrotransferred onto a polyvinylidene fluoride (PVDF) membrane (Millipore, Boston, MA, USA). The membranes were washed with TBS with 0.2% Tween-20 (TBST; Sigma, St. Louis, MO, USA) and then blocked using 5% nonfat milk (Aladdin, Shanghai, China) in TBST for 1 h at room temperature. After this, the membranes were incubated with primary antibodies overnight at 4 °C. Anti-cleaved caspase-3 (9664) was obtained from Cell Signaling Technology (Boston, MA, USA), and the anti-Bcl-2 antibody (12789-1-AP), anti-Bax antibody (60267-1-Ig), anti-Beclin-1 antibody (11306-1-AP), anti-COXIV antibody (11242-1-AP), anti-StAR antibody (12225-1-AP), anti-cytochrome C antibody (10993-1-AP), anti-LHR antibody (19968-1-AP), and anti-β-Actin antibody (66009-1-Ig) were obtained from Proteintech Group (Wuhan, China). The anti-LC-3 antibody (ab48394), anti-p62 antibody (ab56416), and anti-IL-1β antibody (1:500, Abcam, ab4207) were retrieved from Abcam (Cambridge, UK). After washing, the membranes were incubated with horseradish-peroxidase-conjugated secondary antibodies (A0208 or A0216, Beyotime, Haimen, China) at room temperature for 1 h. Finally, the membranes were rinsed with TBST three times (for 10 min each), and the signals were detected using an ECL kit from Beyotime Biotechnology (Haimen, China). 3-Methyladenine (3-MA) (Sigma, St. Louis, MO, USA) (2 mM) was used to inhibit autophagy. Rapamycin (HY-10219, MedChemExpress, Monmouth Junction, NJ, USA) (10 nM) was used to induce autophagy. INF39 (HY-100597, MedChemExpress, Monmouth Junction, NJ, USA) (10 μM) was used to inhibit NLRP3 inflammasome activation.

### 2.6. Intracellular ROS Level Measurements

The level of ROS was determined using a commercial kit (S0033S, Beyotime, Haimen, China). N-acetylcysteine (NAC) (S0077, Beyotime, Haimen, China) (50 mM) was used as a ROS scavenger. Briefly, DCFH-DA was added into the medium at a concentration of 10 μM. After this, cells were incubated in the incubator for 20 min and then washed with serum-free medium 3 times. Finally, the cells were detected under a fluorescence microscope (ZEISS, Oberkochen, Germany).

### 2.7. JC-1 Staining

In order to determine mitochondrial status, we tracked the relative mitochondrial transmembrane potential through 5,5′,6,6′-tetrachloro-1,1′,3,3′-tetraethylimidacarbocyanine iodide (JC-1) staining. The fluorescence of the lipophilic cation JC-1 reversibly changes from green (the monomeric status) to red (the multimeric status) according to the variation in the mitochondrial potential. A JC-1 kit (C2006, Beyotime, Haimen, China) was used to evaluate the mitochondrial status of the Leydig cells according to the methods provided by the manufacturer. Briefly, the cultured Leydig cells were digested into a single cell suspension, collected, and incubated with 10 μg/mL of JC-1 at 37 °C and 5% CO_2_ for 30 min. After washing, the cells were analyzed using a BD FACSymphony A5 system (Becton Dickinson, Franklin, NJ, USA).

### 2.8. ELISA

ELISA kits were used to measure the levels of interleukin-1β (IL-1β) (ab100768, Abcam, Cambridge, UK) and testosterone (ab108666, Abcam, Cambridge, UK) in the serum. The analytical procedures were performed according to the manufacturer’s instructions.

### 2.9. Mitochondrial Isolation

After the isolation of the Leydig cells, the mitochondria were isolated using a commercial kit (Beyotime, Haimen, China). Briefly, the cells were centrifugated and resuspended using isolation reagent. After this, the cells were homogenized under ice-cold conditions and centrifugated at 600× *g* for 5 min, and then the suspension was transferred into a new tube, followed by centrifugation at 11000× *g* and 4 °C for 10 min. We collected the suspension and precipitation for further analysis. Mitochondrial isolation was confirmed according to the expression of the outer membrane marker VDAC.

### 2.10. Transmission Electron Microscopy

After the isolation of the rat Leydig cells, the cells were pelleted through centrifugation. The samples were then fixed using 2.5% (*v*/*v*) glutaraldehyde (P1126, Solarbio, Beijing, China) in PBS (4 °C, pH 7.4, 0.1 M) for 24 h. After this, the specimens were post-fixed in osmium tetroxide (OsO_4_, Aladdin, Shanghai, China) and then embedded into Epon-812 (Aladdin, Shanghai, China). Sections (0.1 mm) were stained using uranyl acetate (Aladdin, Shanghai, China) and lead citrate (Aladdin, Shanghai, China). The images were observed using a HT7700 transmission electron microscope (Hitachi, Tokyo, Japan).

### 2.11. Cell NLRP3 Transfection

NLRP3 siRNA were purchased from Genepharma (Shanghai, China) (sense 5′-GUA UGA ACU CUU GAC CAU UTT-3′, antisense 5′-AAU GGU CAA GAG UUC AUG CTT-3′). Transfections were performed using Lipofectamine 3000 (Invitrogen, Carlsbad, CA, USA) according to the method provided by the manufacturer’s instructions. The medium was replaced 4 h after transfection.

### 2.12. The Statistical Analysis

All experimental values were presented as the mean ± SEM. Significant differences in the mean values within or between treatment groups were evaluated using a one-way analysis of variance, followed by Tukey’s multiple range test. Residual and normality tests of the data, as well as the significance analysis, were performed using GraphPad Software Prism 8.0 (San Diego, CA, USA). A statistically significant difference was inferred at *p* < 0.05.

## 3. Results

### 3.1. Prepubertal Diabetes Is Deleterious to Testicular Development

**Figure 1:**
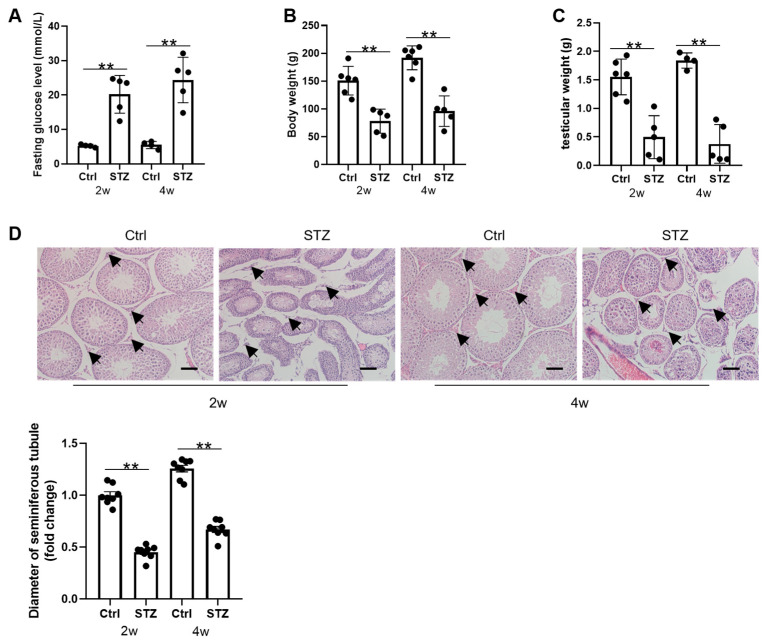
STZ-induced diabetes causes abnormal testicular development. (**A**) Fasting blood glucose levels after 2 or 4 weeks of STZ treatment (*n* = 5). (**B**) Body weights and (**C**) testicular weights of rats after 2 or 4 weeks of STZ treatment (control group: *n* = 6; STZ group: *n* = 5). (**D**) Representative images of H&E staining show testicular morphologies in rats after 2/4 weeks with different treatments. The diameter of the seminiferous tubules was counted using 8 measurements (*n* = 8). The black arrows indicate Leydig cells in the testes. Scale bar = 100 μm. ** *p* < 0.01.

Male prepubertal rat models of diabetes were established via STZ administration. Fasting glucose levels, body weight, and testicular weight were measured at 2 and 4 weeks post-treatment. The diabetic rats exhibited significantly elevated fasting glucose levels at both time points compared to those in the controls, though no significant difference was observed between the 2- and 4-week diabetic groups (Figure 1A). Prepubertal diabetes markedly suppressed their body weight gain and progressively reduced testicular weight with disease duration (Figure 1B,C). The histological analysis (H&E staining) revealed structural impairments in the diabetic testes, including seminiferous tubule atrophy and reduced Leydig cell numbers (Figure 1D). These results indicate that prepubertal diabetes significantly disrupts testicular development in male rats.

### 3.2. Prepubertal Diabetes Attenuates Leydig Cell Differentiation and Improves Cell Apoptosis

**Figure 2:**
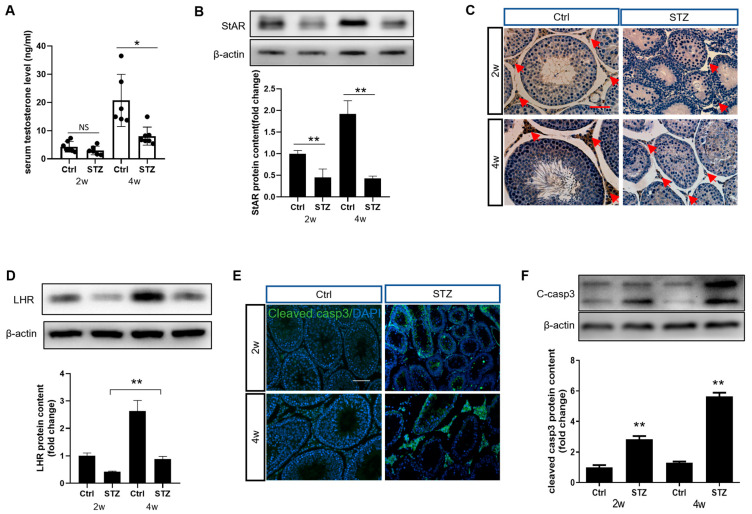
Prepubertal diabetes impairs testicular steroidogenesis and triggers Leydig cell apoptosis. (**A**) Serum testosterone concentrations in rats under diverse treatments (*n* = 6). (**B**) StAR expression levels in Leydig cells from Ctrl or STZ groups at different time points (*n* = 3). (**C**) Immunolocalization of StAR in rat testes. The red arrows indicate Leydig cells in the testes. Scale bar = 100 μm. (**D**) The effect of diabetes on LHR expression (*n* = 3). (**E**) Representative images showing immunofluorescence for cleaved caspase3 expression. Sections were co-stained using DAPI. Scale bar = 100 μm. (**F**) Expression of cleaved caspase3 in different groups (*n* = 3). NS: *p* > 0.05, * *p* < 0.05, ** *p* < 0.01.

Steroidogenic capacity is one of the testis’s most critical functions, supporting somatic development and spermatogenesis. Maturation of the Leydig cells during the prepubertal stage is essential for subsequent steroidogenesis in males. Consistent with this requirement, while testosterone levels showed no significant decrease at 2 weeks post-STZ treatment, they were significantly reduced by 4 weeks after the STZ insult (Figure 2A). However, steroidogenic acute regulatory (StAR) protein expression levels decreased at both time points (Figure 2B and Figure S1 in Supplementary Materials), aligning with the results of immunohistochemical staining (Figure 2C). Furthermore, prepubertal diabetes markedly impaired Leydig cell differentiation, as evidenced by reduced luteinizing hormone receptor (LHR) expression (Figure 2D and Figure S2). Additionally, prepubertal diabetes increased apoptosis, particularly in the Leydig cells (Figure 2E,F, Figures S3 and S4). Other cell death pathways may also be involved in the development of prepubertal diabetes and will be investigated in subsequent studies. These findings indicate that prepubertal diabetes disrupts Leydig cell maturation and significantly compromises testicular function.

### 3.3. Prepubertal Diabetes Induces NLRP3 Expression and an Inflammatory Response in Leydig Cells

**Figure 3:**
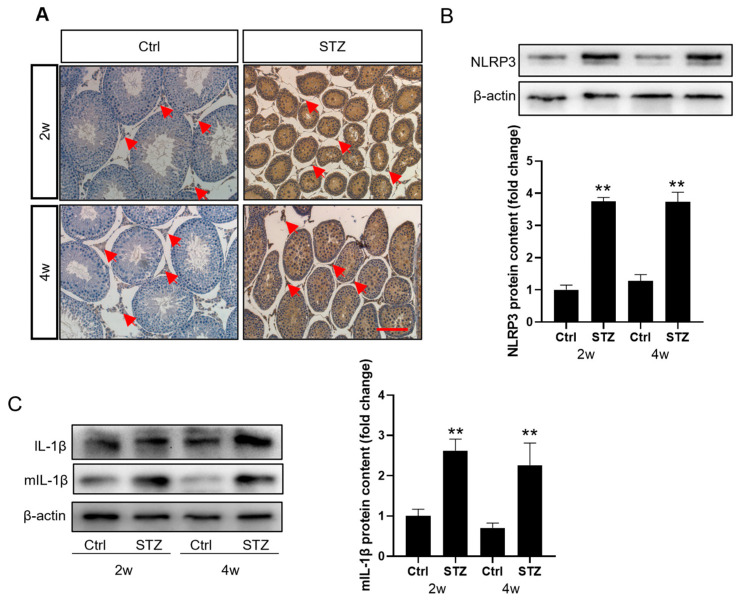
Prepubertal diabetes promotes NLRP3 activation in Leydig cells. (**A**). Immunolocalization of NLRP3 in the rat testes. The red arrows indicate Leydig cells in the testes. Scale bar = 100 μm. (**B**) The Leydig cells were isolated using a Percoll gradient, and the NLRP3 content was detected using a Western blot analysis (*n* = 3). (**C**) The expression of IL-1β and mature IL-1β (mIL-1β) in Leydig cells from normal and STZ-primed rats (*n* = 3). ** *p* < 0.01.

Chronic inflammatory activation is a systemic feature of diabetic organs. This inflammatory response is also observed in the testes of obese males, where it impairs the steroidogenic capacity of the Leydig cells [24]. In the present study, we investigated whether the NLRP3 inflammasome contributed to inflammatory signaling in the diabetic testes. Immunohistochemical staining revealed that the STZ treatment significantly induced NLRP3 expression in the testes, localized to both the Leydig cells and spermatogenic cells (Figure 3A). To assess the magnitude of NLRP3 induction specifically in the Leydig cells, we isolated these cells for a Western blot analysis. The NLRP3 protein levels in the Leydig cells showed a significant increase after the STZ treatment (Figure 3B and Figure S5). Consistently, a significant change in IL-1β activation was observed (Figure 3C and Figure S6). These findings demonstrate that prepubertal diabetes induces NLRP3 expression and an associated inflammatory response in the testes.

### 3.4. Beclin1 Cleavage Is Involved in Diabetes-Induced Autophagy Inhibition in Leydig Cells

**Figure 4:**
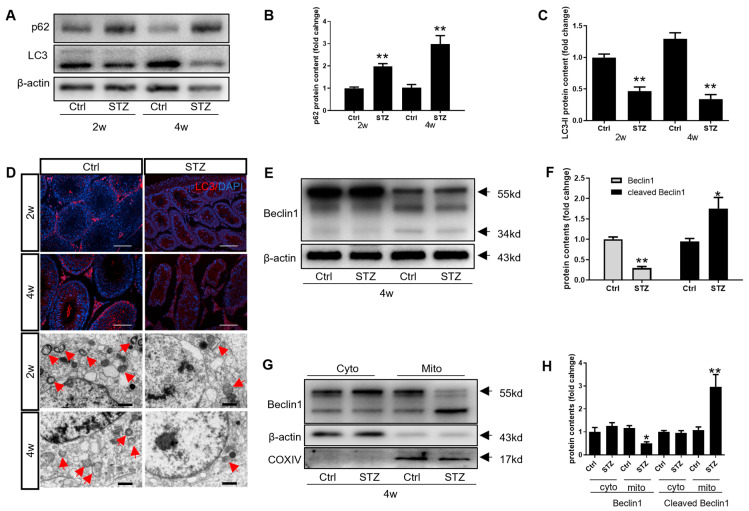
Beclin1 cleavage is engaged in autophagy restraint in Leydig cells. (**A**) The effect of diabetes on the microtubule-associated protein 1 light chain 3 (LC3) and p62 contents were detected through Western blot. (**B**,**C**) Quantitative analysis of protein levels of p62 and LC3-II in (A) (*n* = 3). (**D**) The induction of autophagy was verified through immunofluorescence (scale bar = 100 μm) and TEM observations (scale bar = 10 μm). The red arrow represents the autophagosome. (**E**) The expression of Beclin1 was detected through Western blot. (**F**) Quantitative analysis of protein levels of Beclin1 in its full-length and cleaved forms (*n* = 3). (**G**) The expression of Beclin1 in the cytoplasm and mitochondria was detected using Western blot after mitochondrial isolation. (**H**) Quantitative analysis of protein levels of Beclin1 in its full-length and cleaved forms in the cytoplasm and mitochondria (*n* = 3). * *p* < 0.05, ** *p* < 0.01.

It has been established that the orchestration of autophagy plays essential roles in steroidogenesis and inflammatory responses [20,25]. In the present study, STZ treatment significantly reduced the level of autophagy, as evidenced by the changes in the autophagy-related proteins (Figure 4A–D and Figure S7). The transmission electron microscope (TEM) analysis further confirmed a decline in the autophagosomes within the Leydig cells from the diabetic rats (Figure 4D). Beclin1, a scaffold protein required for autophagosome formation and prominently expressed during autophagy induction [26], was cleaved in the diabetic testicular Leydig cells (Figure 4E,F and Figure S8). Notably, full-length and cleaved Beclin1 exhibited distinct subcellular localization: cleaved Beclin1 primarily localized to the mitochondria, while full-length Beclin1 resided in the cytoplasm (Figure 4G,H and Figure S9). These findings indicate that autophagy is inhibited in the Leydig cells and that Beclin1 cleavage contributes, at least partially, to this regulatory mechanism.

### 3.5. Prepubertal Diabetes Drives a Skew in Mitochondrial Homeostasis in the Leydig Cells by Stagnating Mitophagy

**Figure 5:**
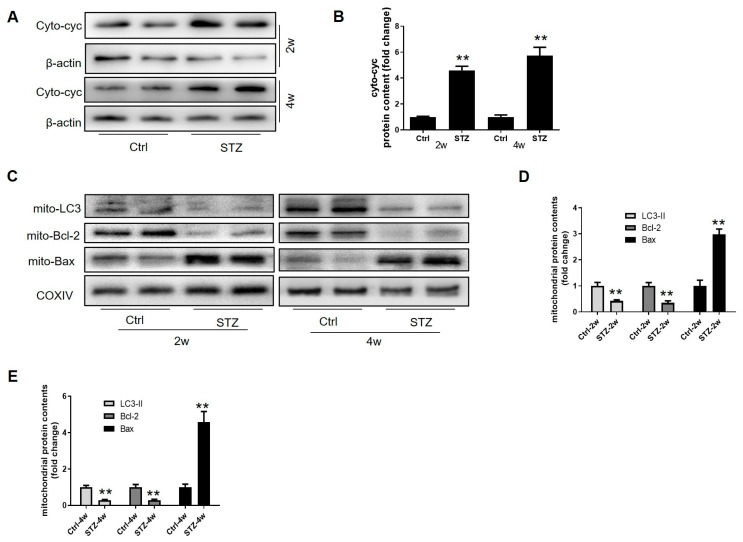
The effect of diabetes on mitochondrial homeostasis in Leydig cells. (**A**) The rats from the Ctrl and diabetic groups were sacrificed 2 weeks or 4 weeks after STZ injection. The mitochondria of the Leydig cells were isolated using a commercial kit (Beyotime, Haimen, China). The level of cytoplasmic cytochrome c was detected using Western blot. (**B**) Quantitative analysis of protein levels of cyto-cyc in Leydig cells (*n* = 3). (**C**) The mitochondria were isolated from the Leydig cells, and the expression of LC3, Bcl-2, and Bax in the mitochondria was detected through Western blot. (**D**) Quantitative analysis of protein levels of LC3, Bcl-2, and Bax in the mitochondria 2 weeks after STZ injection (*n* = 3). (**E**) Quantitative analysis of mitochondrial protein levels of LC3, Bcl-2, and Bax 4 weeks after STZ injection (*n* = 3). ** *p* < 0.01.

The mitochondria are the site of testosterone production, and mitochondrial content is essential for Leydig cell function and survival. We therefore investigated how prepubertal diabetes affected mitochondrial homeostasis in these cells. As expected, diabetes significantly reduced the mitochondrial content, evidenced by decreased COXIV expression, while increasing the cytosolic cytochrome c levels in the Leydig cells (Figure 5A,B and Figure S10). Furthermore, we observed decreased mitochondrial B-cell lymphoma 2 (Bcl-2) and increased Bcl2-associated X (Bax) levels, indicating an impaired mitochondrial balance in the Leydig cells from the diabetic rats (Figure 5C–E and Figure S11). Notably, mitochondria-localized LC3-II was also markedly reduced, suggesting diminished mitophagy (Figure 5C). These findings indicate that prepubertal diabetes disrupts the mitochondrial balance in Leydig cells by attenuating mitophagy.

### 3.6. Autophagy Is Required for Leydig Cell Steroidogenesis and Maturation

**Figure 6:**
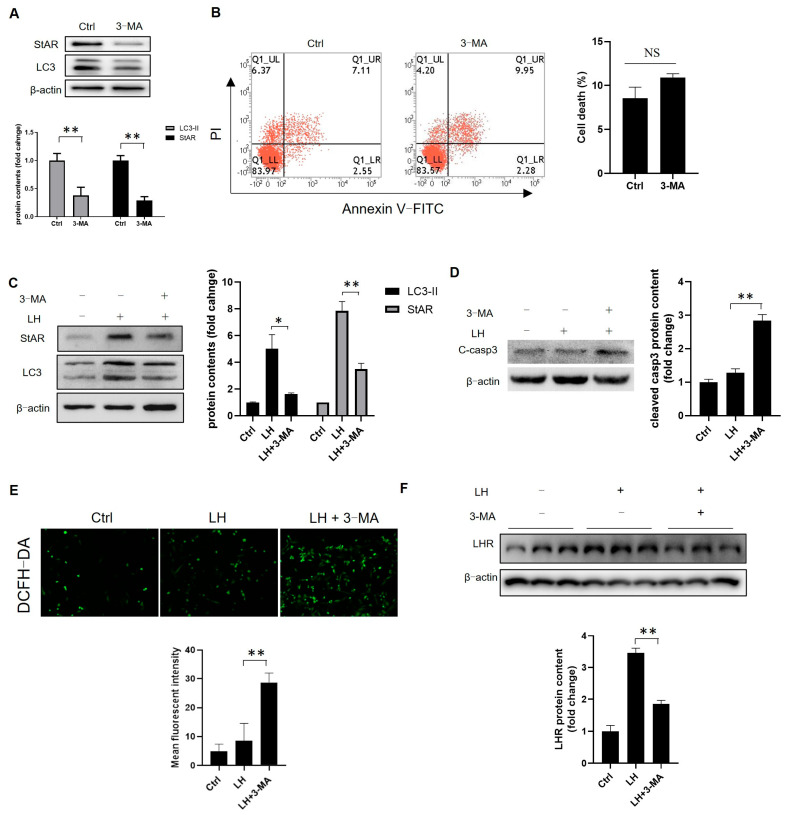
Autophagy is essential to the steroidogenic ability of Leydig cells. (**A**) The expression of StAR and LC3 in Leydig cells with or without 3-MA (10 mM) for 24 h (*n* = 3). (**B**) Apoptosis of the Leydig cells was evaluated using an Annexin-PI staining kit (Aladdin, Shanghai, China) and was detected through flow cytometry (*n* = 3). (**C**) The cells were pretreated with 3-MA (10 μM) for 24 h, followed by LH treatment (5 U/mL) for 72 h. The expression of StAR and LC3 was detected through Western blot (*n* = 3). (**D**) The expression of cleaved caspase3 as detected through Western blot (*n* = 3). (**E**) ROS levels were detected through DCFH-DA staining and observed using a fluorescent microscope (*n* = 3). (**F**) The effect of 3-MA on the LHR expression in the Leydig cells (*n* = 3). NS: *p* > 0.05, * *p* < 0.05, ** *p* < 0.01.

Given the established links between autophagy and steroidogenesis [27], we examined their role in immature Leydig cells from prepubertal rats, which exhibit physiological differences from mature cells. Treating primary prepubertal Leydig cells with the autophagy inhibitor 3-MA for 24 h attenuated the luteinizing hormone (LH)-induced StAR expression (Figure 6A,C, Figures S12 and S13). While 3-MA alone did not affect apoptosis under the basal conditions (Figure 6B), it enhanced caspase-3 cleavage (Figure 6D and Figure S14) and ROS generation (Figure 6E) during LH stimulation. Additionally, autophagy inhibition attenuated Leydig cell differentiation (Figure 6F and Figure S15). Collectively, these results demonstrate that autophagy is essential for Leydig cell maturation and mitigates ROS accumulation and apoptosis.

### 3.7. High Glucose Stagnates Leydig Cell Maturation by Inhibiting Autophagy

**Figure 7:**
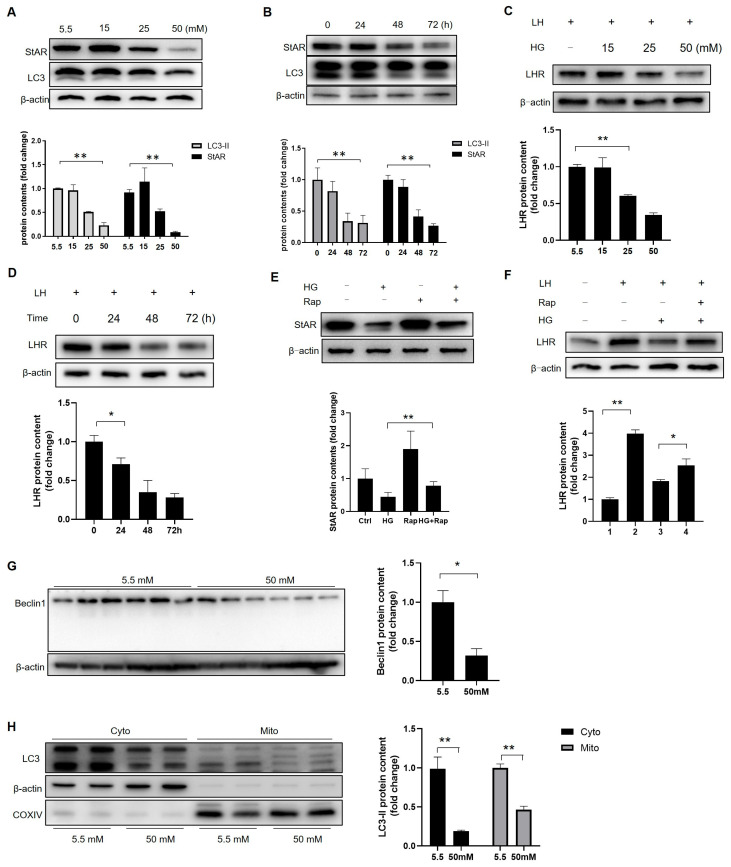
Autophagy is essential to the maturation of Leydig cells. (**A**) The Leydig cells were treated with different concentrations of glucose for 72 h, and the protein content of StAR and LC3 in the cell lysates was analyzed through Western blot (*n* = 3). (**B**) The Leydig cells were exposed to high glucose at a concentration of 50 mM for the indicated times, and the protein levels of StAR and LC3 in the cell lysates were detected through Western blotting (*n* = 3). (**C**) The effect of the concentration of glucose on LHR expression (*n* = 3). (**D**) The expression of LHR after high-glucose (50 mM) treatments for different times (*n* = 3). (**E**) Cells were pretreated with rapamycin (10 nM) for 12 h, followed by high-glucose culture for 72 h (*n* = 3). (**F**) The expression of LHR with or without rapamycin treatment (*n* = 3). (**G**) The expression of Beclin1 with or without HG treatment (*n* = 3). (**H**) The expression of LC3 in the cytoplasm and mitochondria with or without high-glucose treatment (*n* = 3). * *p* < 0.05, ** *p* < 0.01.

The upregulation of StAR and LHR is critical for prepubertal Leydig cell maturation. High glucose (HG) significantly suppressed both the StAR and LHR expression in dose- and time-dependent manners (Figure 7A–D and Figures S16–S19), indicating that hyperglycemia inhibits steroidogenic capacity and maturation. Concomitantly, the autophagy levels decreased under HG, consistent with the in vivo findings (Figure 7A,B), suggesting a potential role of autophagy in maturation. Rapamycin-induced autophagy partially restored StAR and LHR levels (Figure 7E,F, Figures S20 and S21). Although HG compromised the Beclin1 content, we observed no Beclin1 cleavage, suggesting distinct regulatory mechanisms in vitro versus those in vivo (Figure 7G and Figure S22). Both cytosolic and mitochondrial LC3-II declined under HG (Figure 7H and Figure S23), indicating the suppression of general autophagy and mitophagy. Thus, hyperglycemia impairs Leydig cell maturation by suppressing autophagy and mitophagy, albeit through partially divergent mechanisms in vivo.

### 3.8. Facilitating Autophagy Reverses High-Glucose-Induced Mitochondrial Alteration in Immature Leydig Cells

**Figure 8:**
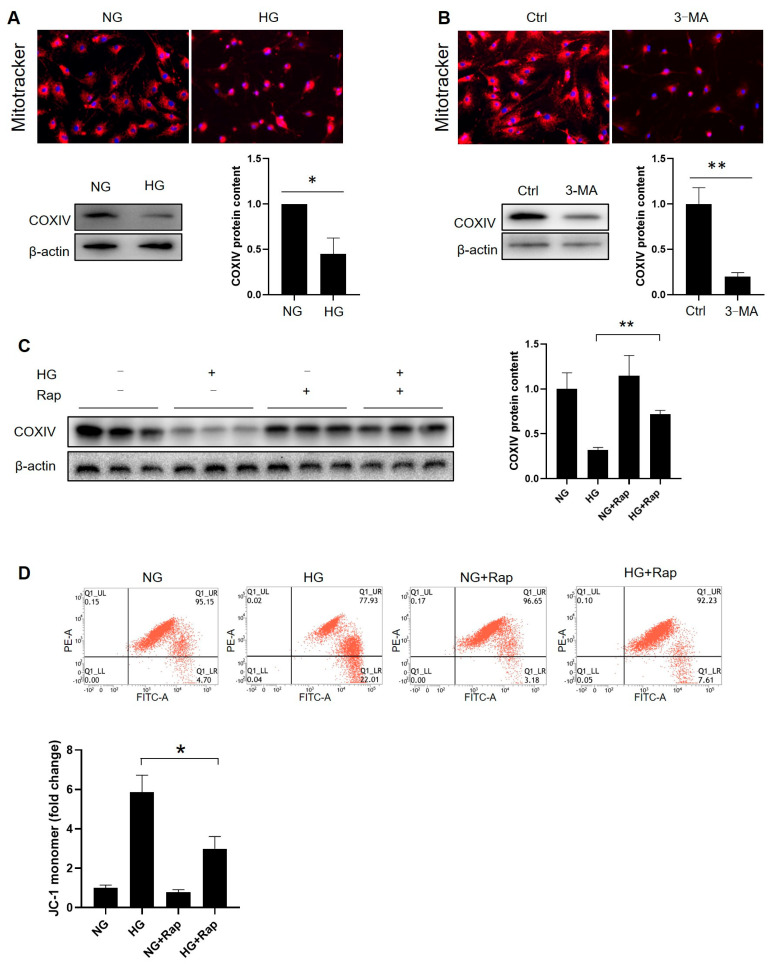
Autophagy is required for mitochondrial homeostasis. (**A**) The Leydig cells were incubated with or without high glucose for 72 h. After that, the cells were subjected to Mitotracker staining or lysed for a protein analysis (*n* = 3). (**B**) The cells were cultured in normal glucose and pretreated with 3-MA (10 mM) at 80% confluence for 24 h followed by high-glucose incubation for 72 h (*n* = 3). After that, cells were subjected to Mitotracker staining or lysed for a protein analysis. (**C**) The cells were pretreated with rapamycin (10 nM) for 12 h, followed by high-glucose treatment for 72 h (*n* = 3). Protein expression of COXIV was analyzed through Western blot. (**D**) The mitochondrial potential was analyzed using a commercial kit, followed by flow cytometry detection (*n* = 3). NG: normal glucose; HG: high glucose; Rap: rapamycin. * *p* < 0.05, ** *p* < 0.01.

Since HG disrupts mitochondrial metabolism and homeostasis [28], we investigated its role in regulating steroidogenesis via the mitochondrial balance in prepubertal Leydig cells. Mitotracker staining and reduced COXIV expression confirmed that HG markedly inhibits the mitochondrial content (Figure 8A and Figure S24). Similarly, 3-MA-mediated autophagy inhibition reduced the mitochondrial content (Figure 8B and Figure S25). Conversely, rapamycin partially restored the COXIV levels (Figure 8C and Figure S26), indicating that autophagy regulates the mitochondrial content. Rapamycin also prevented the HG-induced loss of the mitochondrial membrane potential (Figure 8D). These findings establish hyperglycemia as a key factor reducing the mitochondrial content and membrane potential in diabetic Leydig cells, partly through attenuated autophagy.

### 3.9. The Induction of Autophagy Abrogates High-Glucose-Induced NLRP3 Activation in Leydig Cells

**Figure 9:**
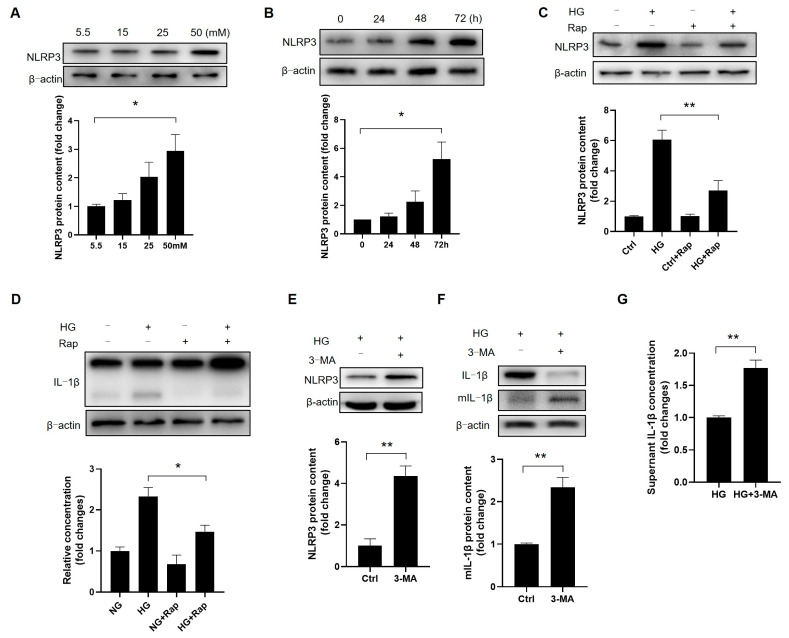
Autophagy is a suppressor of the NLRP3-mediated inflammatory response. (**A**) The Leydig cells were incubated in media with different glucose concentrations for 72 h, and the protein expression was detected using Western blotting (*n* = 3). (**B**) The Leydig cells were incubated in high-glucose medium (50 mM) for different time points, and the protein expression was detected through Western blotting (*n* = 3). (**C**) To verify the role of autophagy in NLRP3 activation, the cells were pretreated with or without rapamycin (10 nM) for 12 h, followed by high-glucose treatment for 72 h (*n* = 3). (**D**) The expression of IL-1β was detected through Western blotting (*n* = 3). (**E**–**G**) The Leydig cells were pretreated with 3-MA (10 mM) for 24 h, followed by high-glucose treatment for 72 h, and the protein expression was detected through Western blotting (*n* = 3). * *p* < 0.05, ** *p* < 0.01.

Given the NLRP3 expression and inflammatory activation in the Leydig cells, we tested whether HG elevates NLRP3. Treating the cells with 5.5–50 mM glucose for 72 h revealed that 50 mM HG significantly increased NLRP3 (Figure 9A and Figure S27). Time-course experiments confirmed this effect (Figure 9B and Figure S28), implicating HG in NLRP3 activation. As autophagy regulates the NLRP3 inflammasome [29], we found that rapamycin partially suppressed NLRP3 and IL-1β activation (Figure 9C,D, Figures S29 and S30). Conversely, 3-MA enhanced the NLRP3 expression and IL-1β maturation (Figure 9E–G, Figures S31 and S32), demonstrating autophagy’s essential role in suppressing NLRP3-mediated inflammation under hyperglycemia. Thus, autophagy inhibits NLRP3 and prevents NLRP3-driven inflammatory responses in the Leydig cells during hyperglycemia.

### 3.10. ROS Hamper Leydig Cell Steroidogenesis and Maturation by Fortifying NLRP3-Mediated Inflammation

**Figure 10:**
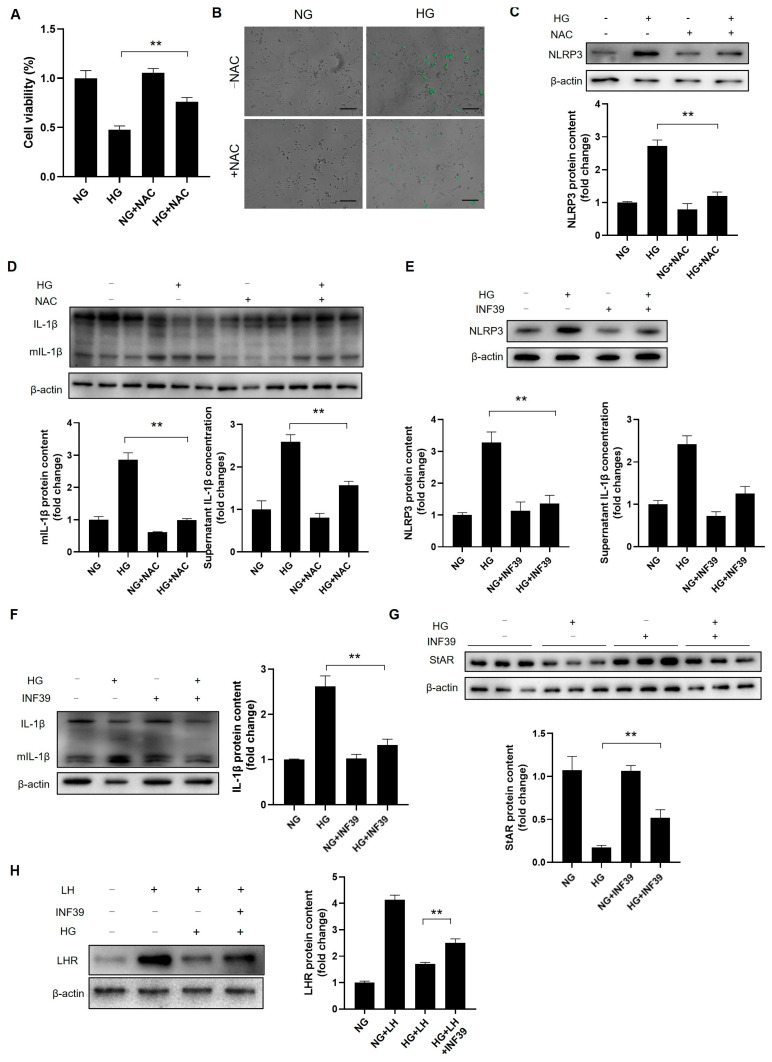
ROS-activated NLRP3 attenuates steroidogenic ability in Leydig cells. (**A**) Leydig cells were pretreated with NAC (50 mM) for 12 h, followed by high-glucose treatment for 72 h (*n* = 3). The viability of the cells was evaluated using a CCK8 kit (Beyotime, Haimen, China). (**B**) The effect of NAC on ROS generation. Scale bar = 200 μm. (**C**) The effect of NAC on the NLRP3 expression (*n* = 3). (**D**) The effect of NAC treatment on IL-1β activation (*n* = 3). (**E**) The expression of NLRP3 after the INF39 treatment (10 μM) (*n* = 3). Cells were cultured in high glucose with or without the presence of INF39 for 72 h. (**F**) The effect of the INF39 treatment on IL-1β activation (*n* = 3). (**G**) The expression of StAR after the INF39 treatment (*n* = 3). (**H**) The effect of the INF39 treatment on progenitor Leydig cell maturation (*n* = 3). NG: normal glucose; HG: high glucose; NAC: N-acetylcysteine; LH: luteinizing hormone. ** *p* < 0.01.

ROS critically impact cell function, survival, and inflammation in diabetes [30]. HG significantly reduced Leydig cell viability while increasing the ROS accumulation; both effects were reversed by the antioxidant NAC (Figure 10A,B). NAC also reversed HG-induced NLRP3 upregulation and IL-1β activation (Figure 10C,D, Figures S33 and S34), indicating that ROS act upstream of NLRP3/IL-1β. Inhibiting NLRP3 with INF39 reduced the HG-induced NLRP3 expression and IL-1β activation (Figure 10E,F, Figures S35 and S36), confirming that NLRP3 drives inflammatory responses in Leydig cells under HG.

As inflammation impairs steroidogenesis in adult Leydig cells [31], we hypothesized that NLRP3 elevation affects immature cells. Inhibiting NLRP3 with INF39 ameliorated the HG-induced decrease in StAR and increased the LHR expression (Figure 10G,H, Figures S37 and S38), identifying NLRP3 as a key regulator of steroidogenesis and maturation. Together, these results demonstrate that HG-induced ROS accumulation activates NLRP3, thereby promoting inflammation and impairing steroidogenesis and maturation in immature Leydig cells.

## 4. Discussion

**Figure 11:**
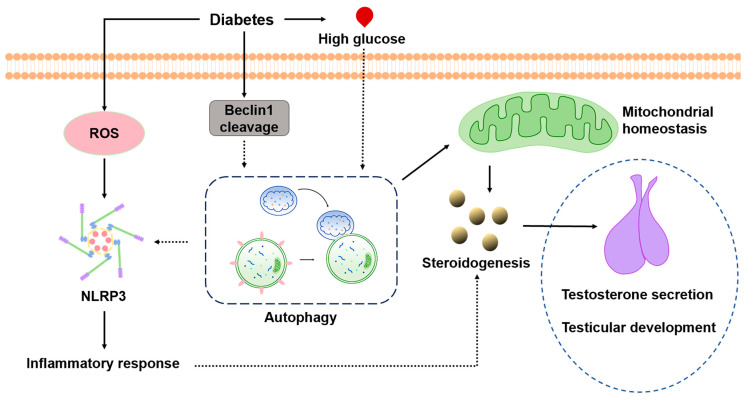
A schematic illustrating how prepubertal diabetes stagnates testicular development by skewing autophagy homeostasis in Leydig cells.

Diabetes has become prevalent across all age groups and is increasingly affecting younger populations. Substantial evidence indicates that the progression of diabetes exerts detrimental effects on the male reproductive system, ultimately contributing to an infertile phenotype [4,11,32]. In this study, we demonstrate that prepubertal diabetes significantly impedes the establishment of steroidogenic capacity and normal testicular development. Furthermore, we show that impaired autophagy regulation underlies dysregulated mitochondrial homeostasis and elevated inflammation in the Leydig cells (Figure 11).

It is well established that the steroidogenic capacity of the Leydig cells increases progressively during prepubertal maturation, enabling somatic development and the acquisition of fertility [33]. Beyond its role in reproduction, testosterone also supports muscle and bone development [34]. However, Leydig cell function and testicular development can be significantly compromised by various diseases, particularly diabetes [35]. Diabetes is a major contributor to morbidity and mortality; its complications adversely affect nearly all organ systems [2]. Ample evidence indicates the deleterious effects of diabetes on male testicular function in both humans and rodent models [4,9]. Nevertheless, few studies have investigated the impact of diabetes on prepubertal testicular development, and its underlying mechanisms remain largely unknown. In this study, we found that prepubertal diabetes significantly disrupts normal testicular physiology, manifesting as an impaired testicular structure, a reduced steroidogenic capacity, decreased testosterone levels, and increased apoptosis. Notably, this study is the first to demonstrate the detrimental effect of diabetes on Leydig cell differentiation, a critical process for acquiring the steroidogenic capacity necessary to produce sufficient testosterone for testicular development.

The mitochondria are the primary sites of testosterone production; mitochondrial dysfunction leads to metabolic disorders in Leydig cells, consequently impairing steroidogenesis [36]. Mitochondrial reprogramming is also involved in Leydig cell differentiation. Nutrients, including glucose, are crucial physiological factors influencing cell differentiation and developmental decisions in mammals. Substantial evidence indicates that hyperglycemia is a key causative factor of mitochondrial dysfunction across various mammalian cell types [37,38]. Therefore, maintaining physiological glucose levels is essential for cellular metabolic homeostasis. Mechanistic studies show that a disrupted mitochondrial balance under hyperglycemic conditions is strongly associated with oxidative stress [39], characterized by an imbalance between ROS generation and elimination. While ROS are normal byproducts of mitochondrial metabolism, their levels can be elevated further by exogenous stimuli and endogenous molecules released from damaged cells. Previous studies indicate that increased ROS levels significantly contribute to hyperglycemia-induced cell death and inflammation [40]. Consistently, mitochondrial morphological abnormalities and elevated ROS generation have also been observed in rat Leydig cells under diabetic conditions [41]. In this study, we found that hyperglycemia adversely affects multiple aspects of Leydig cell physiology, including cell viability, mitochondrial content, mitochondrial membrane potential, steroidogenic capacity, inflammation, and maturation. However, ROS inhibition partially reversed these impairments in cell viability and inflammation. Further investigation demonstrated that NLRP3 mediates hyperglycemia-induced inflammation, and NLRP3 inhibition attenuated IL-1β activation and the decline in the steroidogenic capacity in the Leydig cells. These findings align with previous evidence that inflammation is deleterious to Leydig cell steroidogenesis [18]. Additionally, our study revealed that ROS-induced NLRP3 activation also impedes Leydig cell differentiation. Therefore, insufficient testosterone levels likely result from two factors: a decreased steroidogenic capacity in existing Leydig cells and impaired maturation of immature Leydig cells.

Autophagy, an evolutionarily conserved metabolic mechanism in eukaryotes, targets cellular components for lysosomal degradation, providing energy to maintain metabolic balance, prevent cell death, and mitigate inflammation under stress. Autophagic activation is fundamental for cellular homeostasis, acting as a metabolic rheostat against stressful insults. Autophagy dysregulation is strongly linked to numerous diseases, including cancer, diabetes, and cardiovascular disorders [16]. Interestingly, steroidogenic cells exhibit higher basal autophagy levels than those in other cell types, playing a critical role in steroidogenesis in both sexes [27,42]. However, the precise role of autophagy in regulating testosterone production remains somewhat controversial. For instance, some studies suggest that autophagy inhibition increases the size and number of lipid droplets in rat Leydig cells [43], while findings in mouse Leydig cells indicate that autophagy attenuation impedes cholesterol uptake and lipid droplet formation [20]. Nevertheless, autophagy dysregulation is detrimental to Leydig cell function. Importantly, beyond its role in lipid droplet catabolism, autophagy also influences testosterone synthesis by regulating mitochondrial homeostasis. The mitochondria are the primary source of ROS, and excessive ROS production can impair mitochondrial function further [44]. Autophagy protects the mitochondrial balance by controlling ROS levels. In this study, we show that autophagy is significantly reduced in Leydig cells from diabetic rats, and hyperglycemia is a key factor driving this effect. We also investigated the role of autophagy in the mitochondrial balance under diabetic conditions. Autophagy inhibition clearly disrupted mitochondrial homeostasis, affecting the mitochondrial content, membrane potential, and ROS levels. Interestingly, we observed Beclin1 cleavage in the Leydig cells from diabetic rats but not under hyperglycemia in vitro. These findings suggest that autophagy dysregulation plays a critical role in diabetes-induced testicular impairment, although the regulatory mechanisms may differ between in vivo and in vitro models.

The interplay between autophagy and inflammation has been elucidated in recent years, highlighting autophagy as a key mechanism counteracting excessive inflammatory responses [45]. In this study, we demonstrate that diabetes/hyperglycemia reduces autophagy in Leydig cells, leading to a diminished mitochondrial content and elevated ROS. Furthermore, we show that autophagy is an essential regulator of NLRP3 expression and inflammation. These results suggest that regulating oxidative stress levels and improving autophagic homeostasis are potential targets for treating testicular damage in prepubertal diabetes.

It has long been recognized that autophagy is highly active and crucial during mammalian cell differentiation, acting as a rapid response to hormonal stimuli [46]. Recent studies using systemic and tissue-specific ATG-knockout mouse models have expanded our understanding of autophagy’s roles in mammalian differentiation and development. Cellular differentiation inevitably involves functional transformation, requiring significant intracellular reprogramming of gene expression and metabolism. Indeed, Leydig cells progressively acquire a steroidogenic capacity and undergo morphological changes from mesenchymal-like to round during maturation [47]. While autophagy’s role in Leydig cell steroidogenesis is established, its impact on differentiation remains unclear. Recent evidence indicates increasing autophagy levels during mouse Leydig cell development [20], further suggesting its importance for maturation. In this study, we found that autophagy increases during Leydig cell maturation, a process attenuated by hyperglycemia. Autophagy inhibition also stalled differentiation. Therefore, we conclude that autophagy disruption is the primary mechanism underlying testicular impairment in prepubertal diabetic rats.

## 5. Conclusions

In summary, this study demonstrates that prepubertal diabetes severely impairs Leydig cell differentiation and testicular development by disrupting autophagy, which leads to mitochondrial dysfunction and elevated ROS. Furthermore, we show that ROS act upstream of NLRP3 activation and inflammation in Leydig cells. Collectively, our findings indicate that oxidative stress and inflammation, induced by impaired autophagy, constitute the underlying mechanisms of testicular dysfunction in diabetic rats.

## Data Availability

The data presented in this study are available on request from the corresponding author.

## Supplementary Materials

The following supporting information can be downloaded at https://www.mdpi.com/article/10.3390/cells14171376/s1. Figure S1: The uncropped counterpart of Figure 2B; Figure S2: The uncropped counterpart of Figure 2D; Figure S3: Representative images (negative control) showing immunofluorescence for cleaved caspase3; Figure S4: The uncropped counterpart of Figure 2F; Figure S5: The uncropped counterpart of Figure 3B; Figure S6: The uncropped counterpart of Figure 3C; Figure S7: The uncropped counterpart of Figure 4A; Figure S8: The uncropped counterpart of Figure 4E; Figure S9: The uncropped counterpart of Figure 4G; Figure S10: The uncropped counterpart of Figure 5A; Figure S11: The uncropped counterpart of Figure 5C; Figure S12: The uncropped counterpart of Figure 6A; Figure S13: The uncropped counterpart of Figure 6C; Figure S14: The uncropped counterpart of Figure 6D; Figure S15: The uncropped counterpart of Figure 6F; Figure S16: The uncropped counterpart of Figure 7A; Figure S17: The uncropped counterpart of Figure 7B; Figure S18: The uncropped counterpart of Figure 7C; Figure S19: The uncropped counterpart of Figure 7D; Figure S20: The uncropped counterpart of Figure 7E; Figure S21: The uncropped counterpart of Figure 7F; Figure S22: The uncropped counterpart of Figure 7G; Figure S23: The uncropped counterpart of Figure 7H; Figure S24: The uncropped counterpart of Figure 8A; Figure S25: The uncropped counterpart of Figure 8B; Figure S26: The uncropped counterpart of Figure 8C; Figure S27: The uncropped counterpart of Figure 9A; Figure S28: The uncropped counterpart of Figure 9B; Figure S29: The uncropped counterpart of Figure 9C; Figure S30: The uncropped counterpart of Figure 9D; Figure S31: The uncropped counterpart of Figure 9E; Figure S32: The uncropped counterpart of Figure 9F; Figure S33: The uncropped counterpart of Figure 10C; Figure S34: The uncropped counterpart of Figure 10D; Figure S35: The uncropped counterpart of Figure 10E; Figure S36: The uncropped counterpart of Figure 10F; Figure S37: The uncropped counterpart of Figure 10G; Figure S38: The uncropped counterpart of Figure 10H; Table S1: Primer sequences for real-time PCR.
