# Supplementary material for: Prepubertal Diabetes Stagnates Testicular Development by Skewing Autophagy Homeostasis in Leydig Cells
Source: Cells. 2025 Sep 4;14(17):1376. doi: 10.3390/cells14171376 (PMC12428006; doi:10.3390/cells14171376)
Supplement: Supplementary file 1 [file cells-14-01376-s001.zip › cells-3748334-supplementary.pdf]

Supplementary Materials

Prepubertal Diabetes Stagnates Testicular Development by Skewing Autophagy Homeostasis in Leydig Cells

Zonghao Tang and Youkun Zheng *

Basic Medicine Research Innovation Center for Cardiometabolic Diseases, Ministry of Education, Southwest Medical University, Luzhou 646000, China

*Correspondence: Youkun Zheng, E-mail addresses: zyktmx@163.com

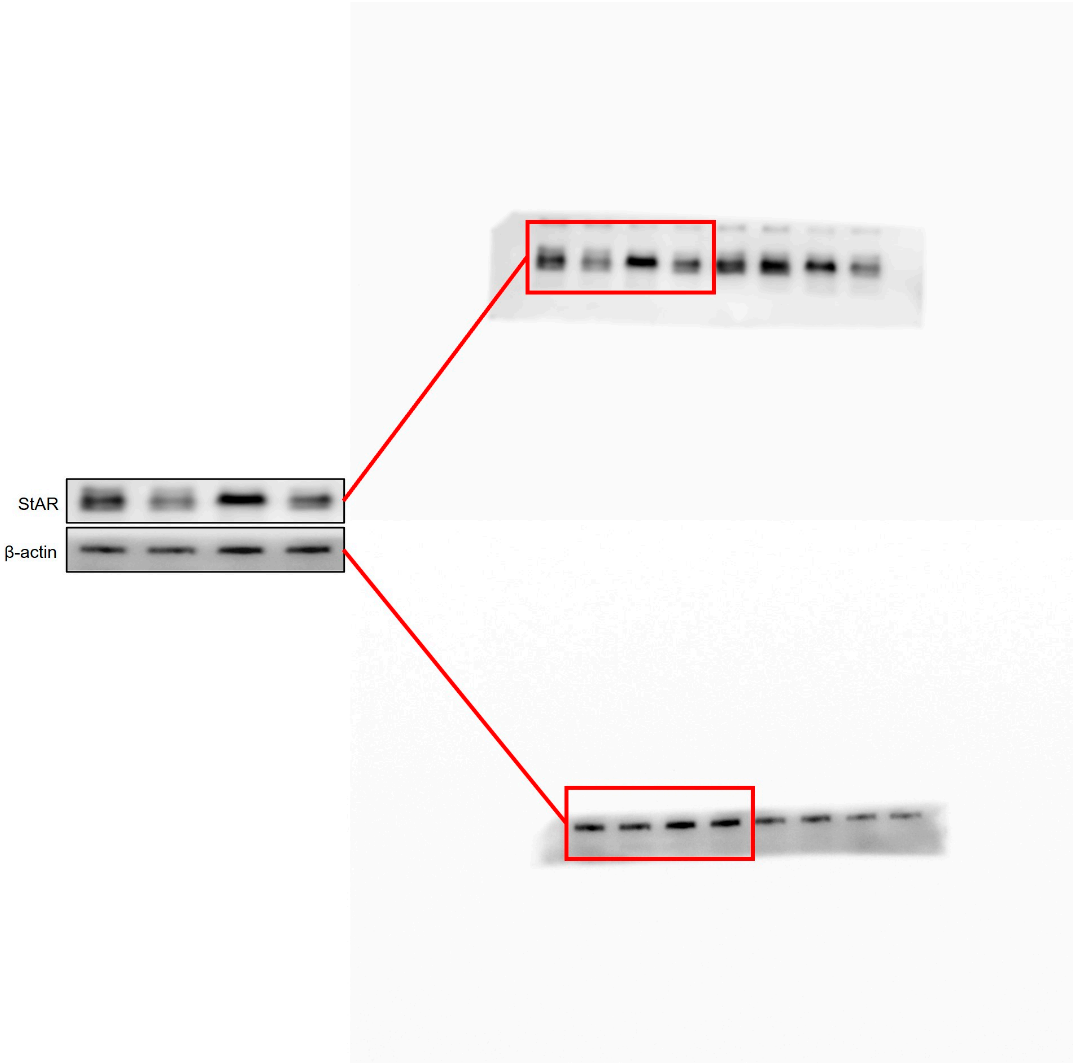

Figure S1. The uncropped counterpart of Figure 2B.

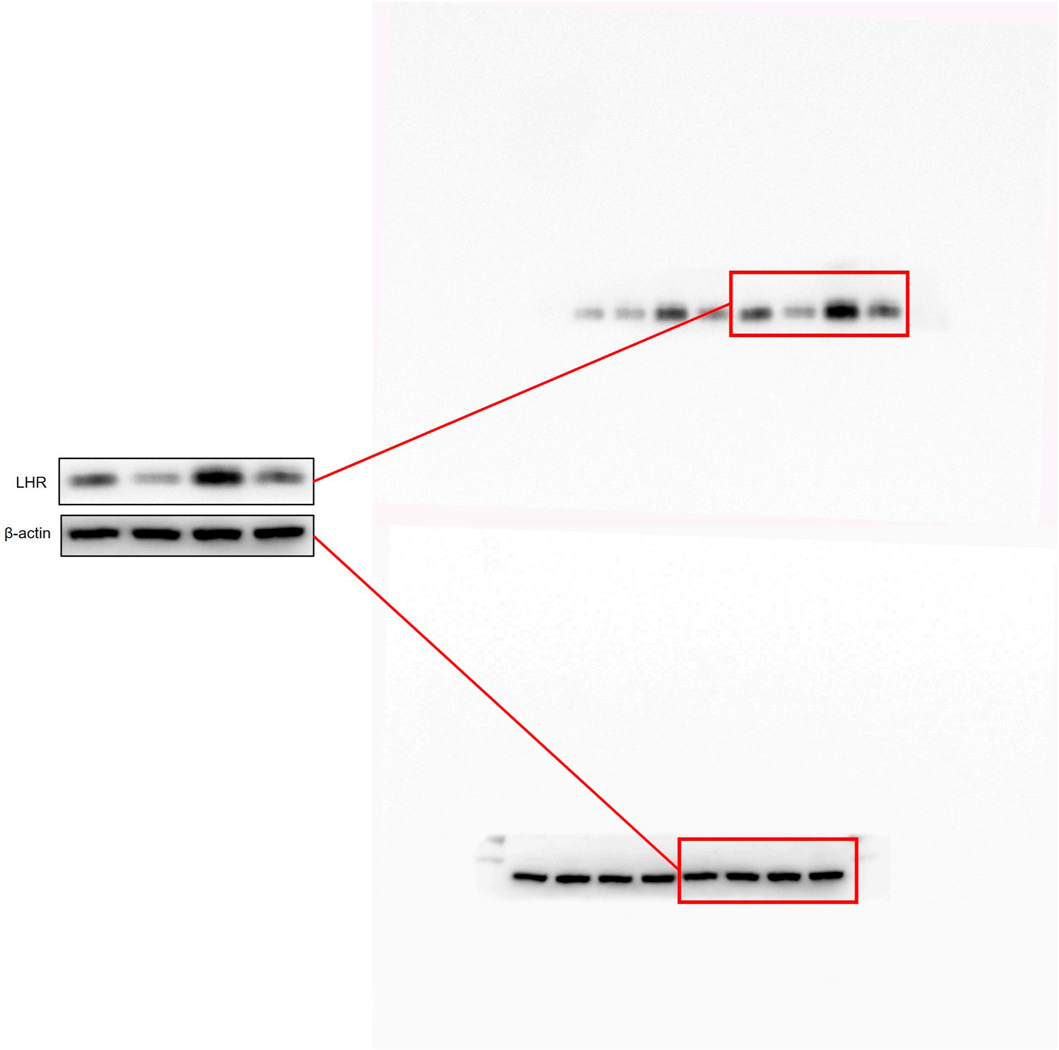

Figure S2. The uncropped counterpart of Figure 2D.

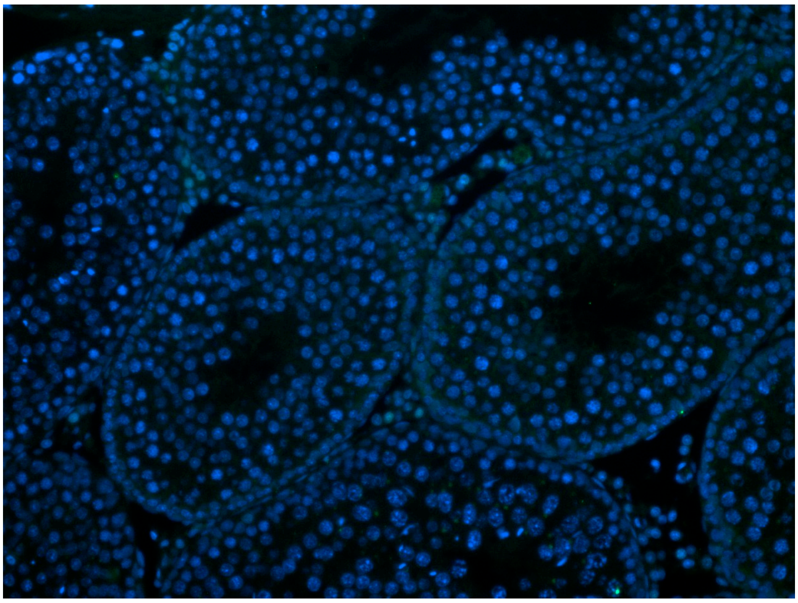

Figure S3. Representative images (negative control) showing immunofluorescence for cleaved caspase3.

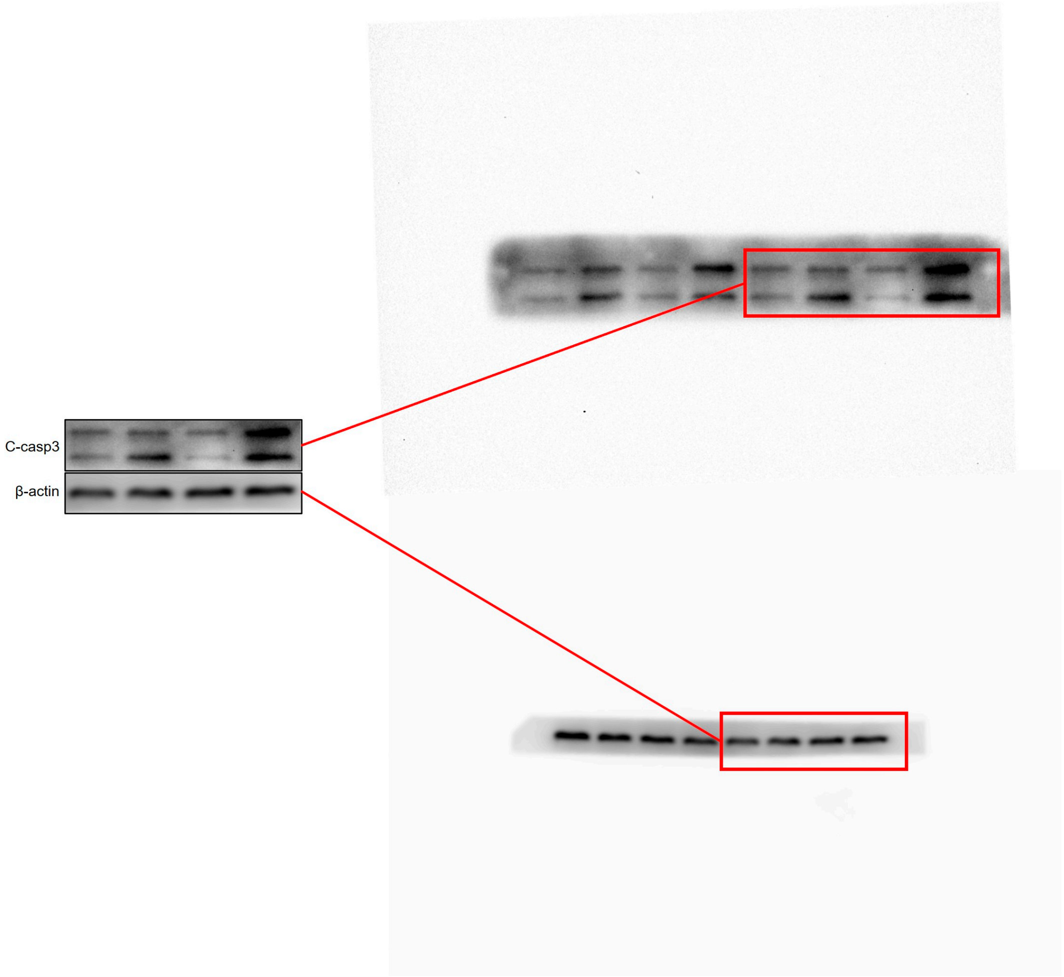

Figure S4. The uncropped counterpart of Figure 2F.

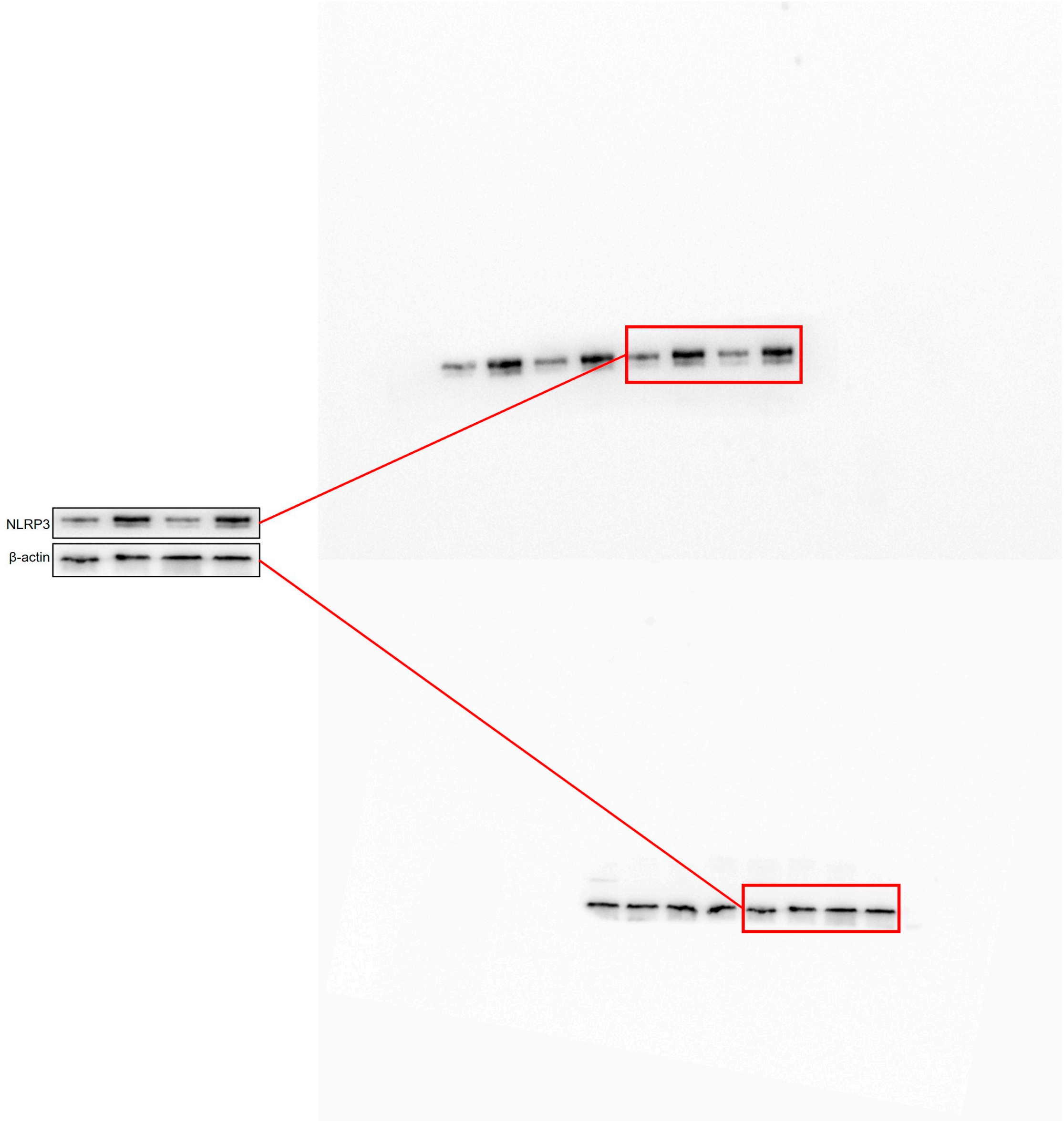

Figure S5. The uncropped counterpart of Figure 3B.

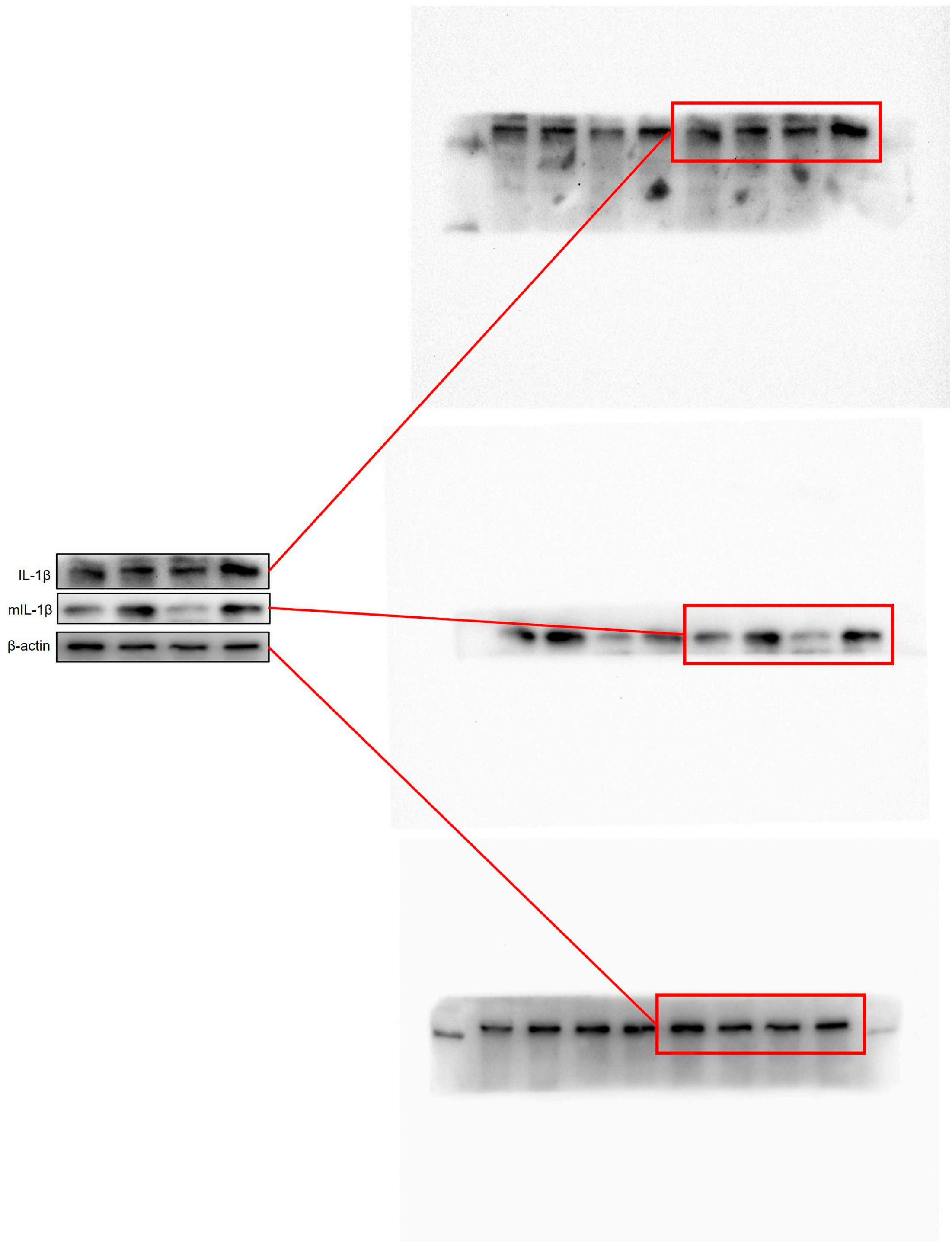

Figure S6. The uncropped counterpart of Figure 3C.

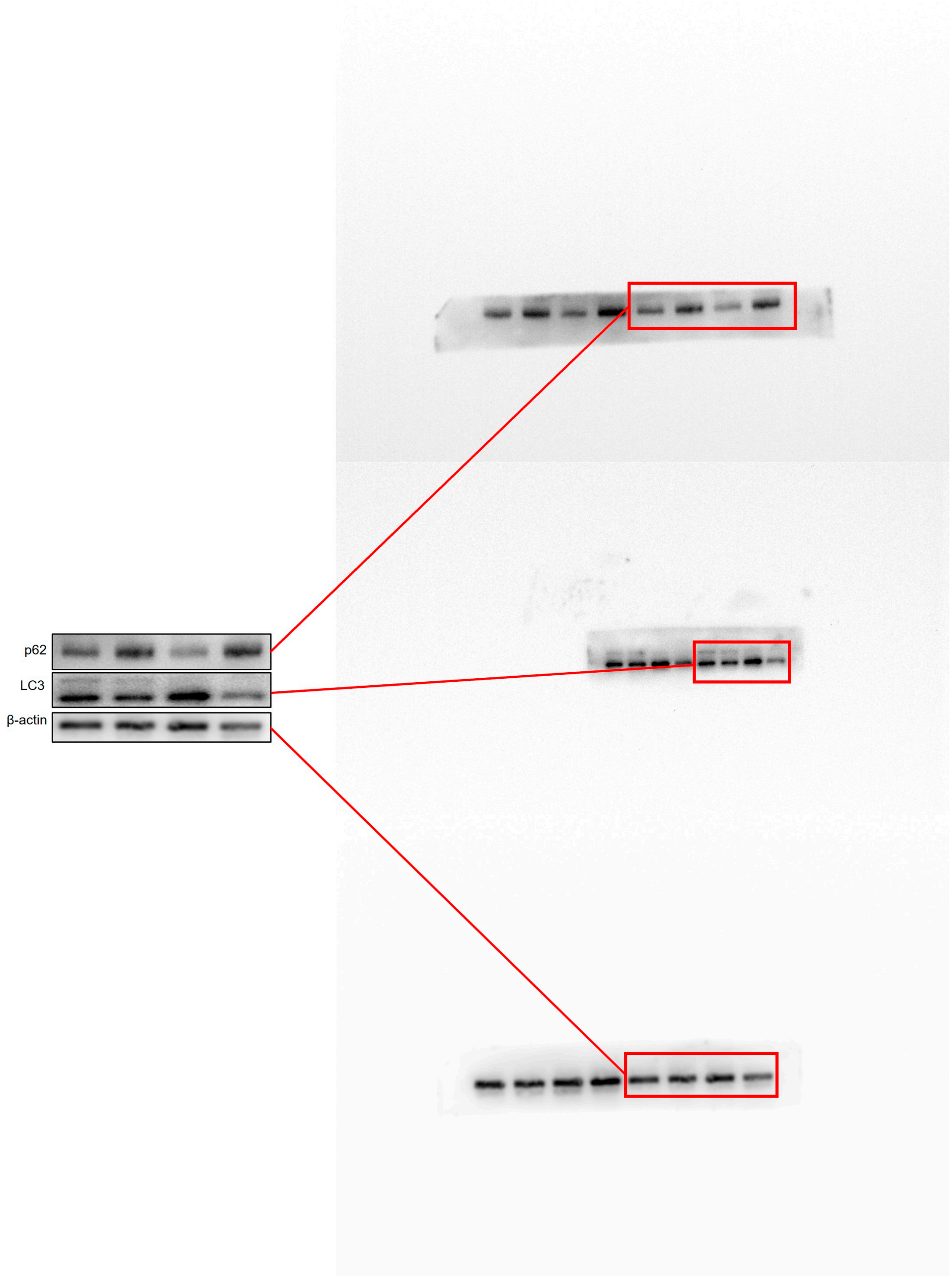

Figure S7. The uncropped counterpart of Figure 4A.

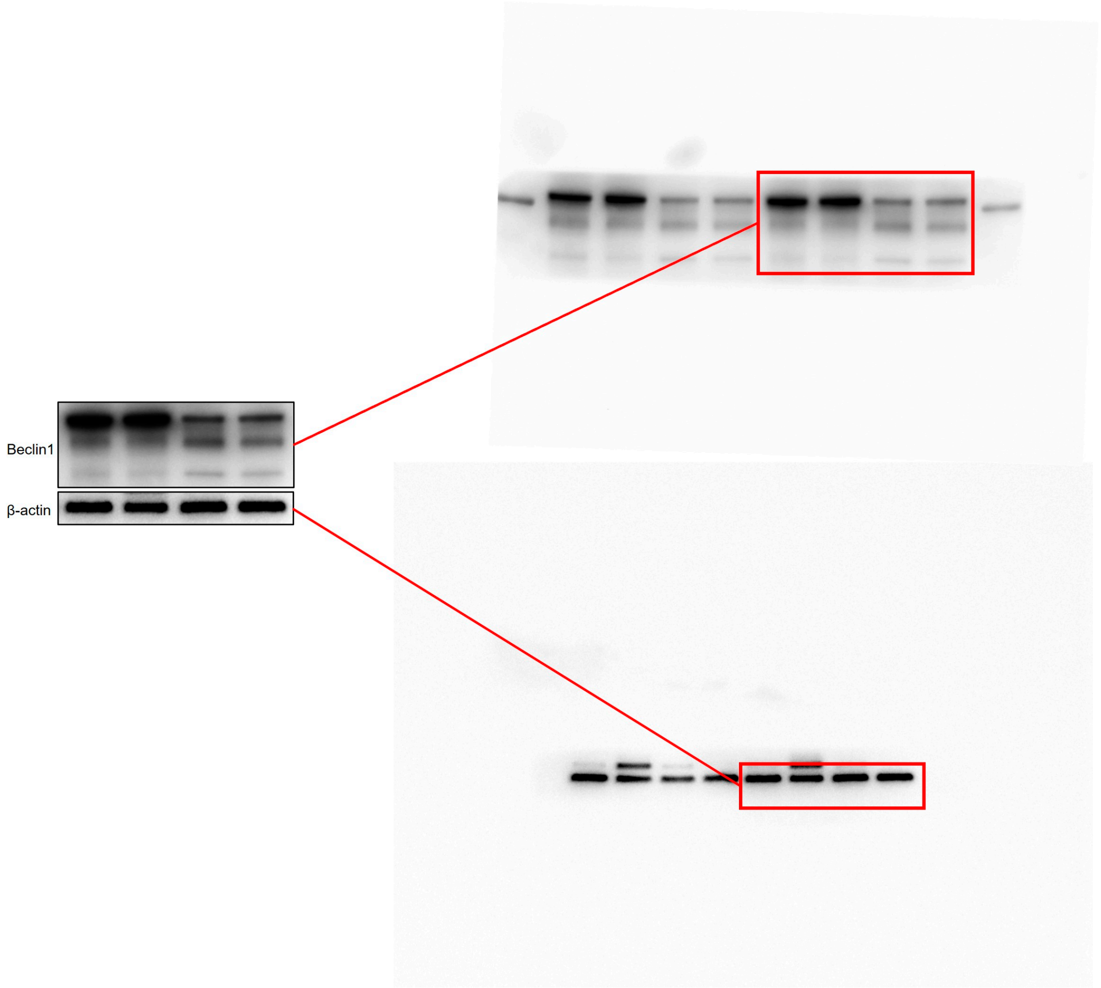

Figure S8. The uncropped counterpart of Figure 4E.

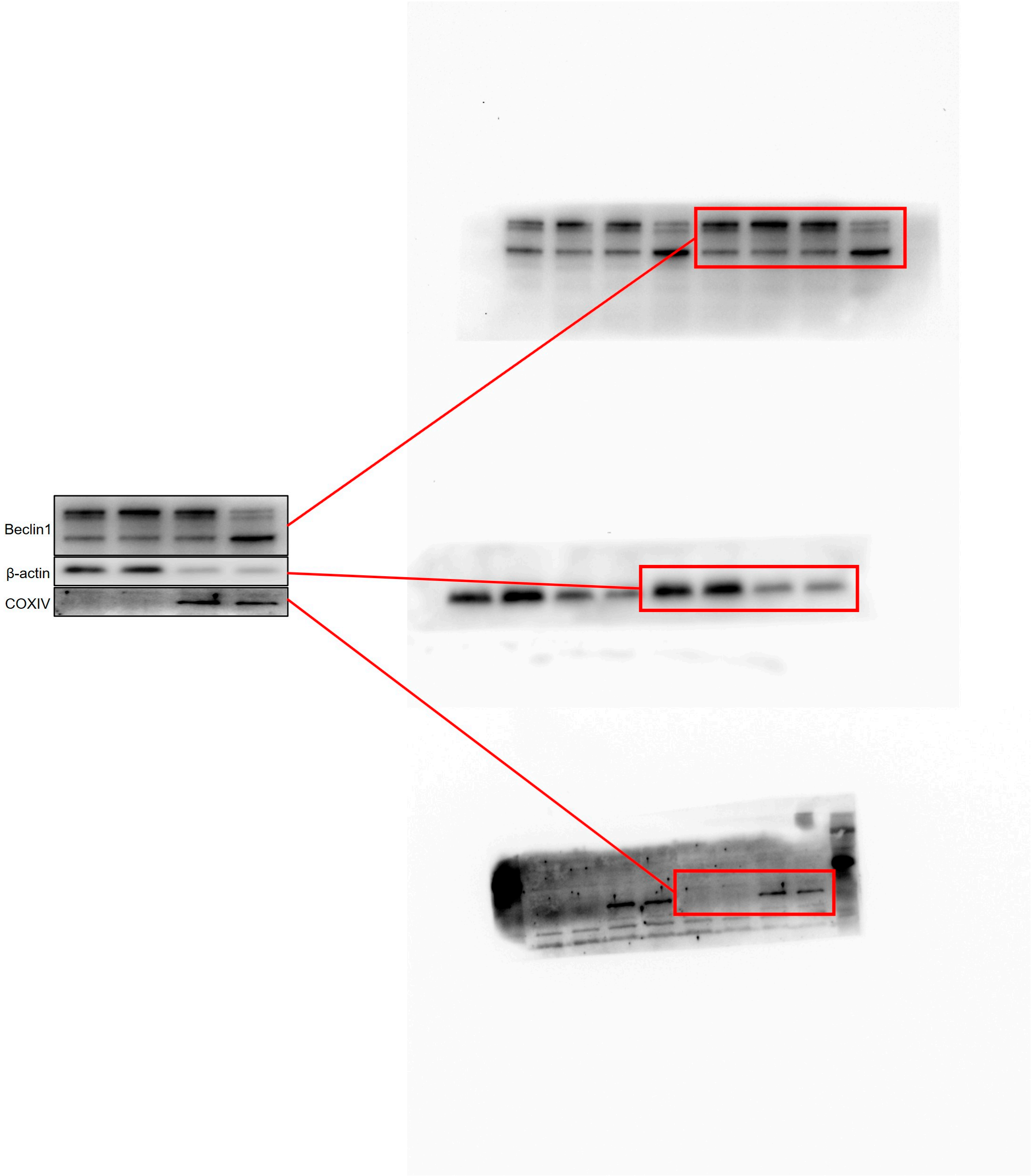

Figure S9. The uncropped counterpart of Figure 4G.

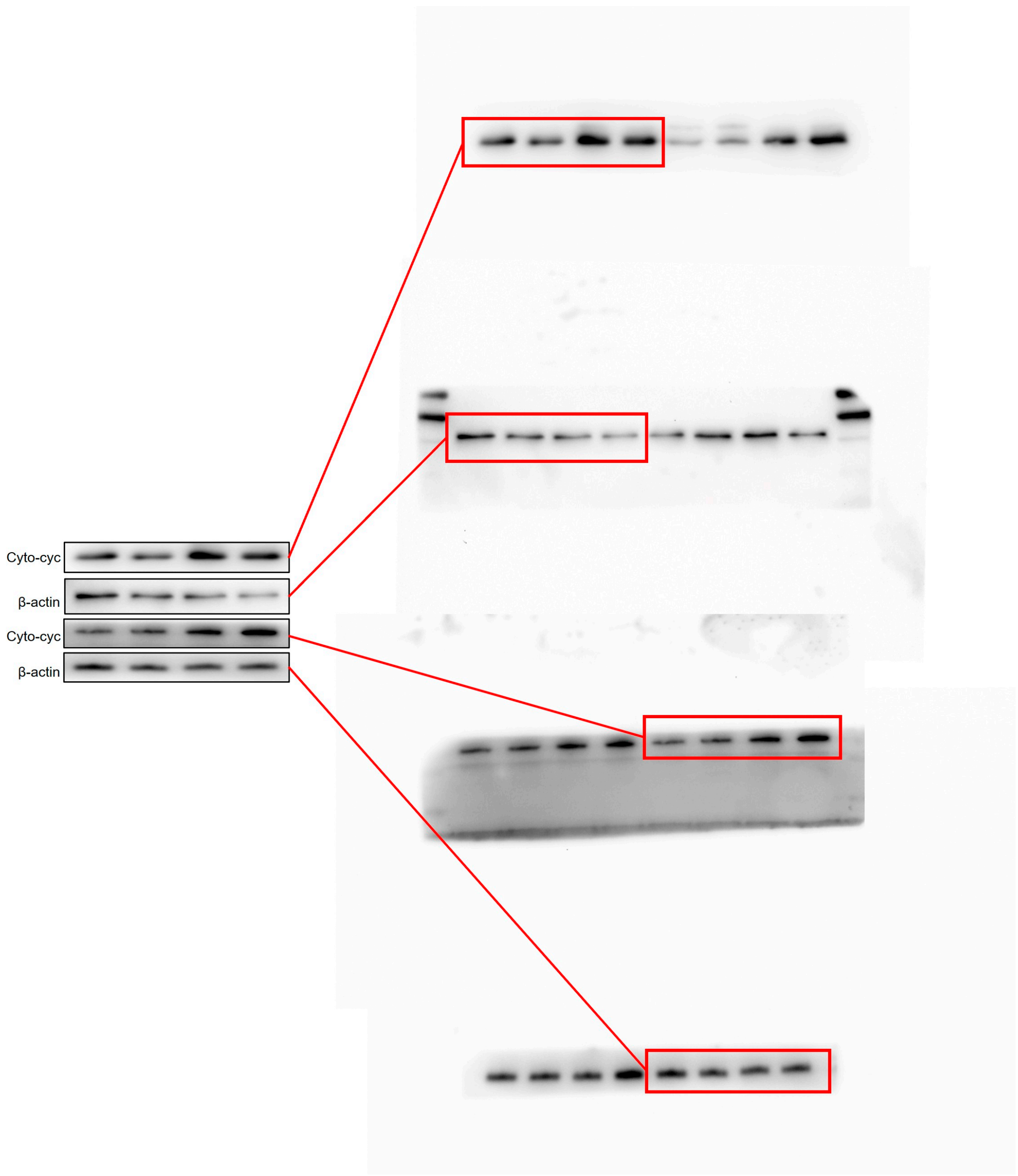

Figure S10. The uncropped counterpart of Figure 5A.

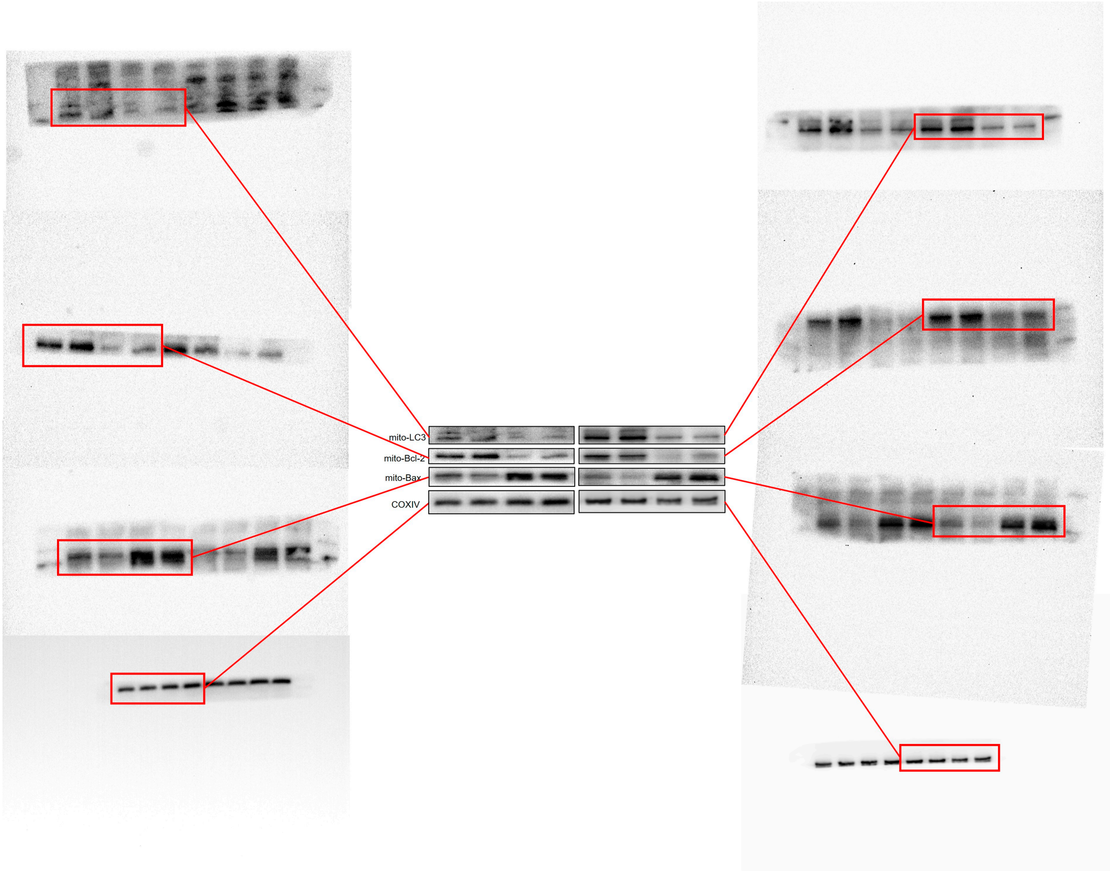

Figure S11. The uncropped counterpart of Figure 5C.

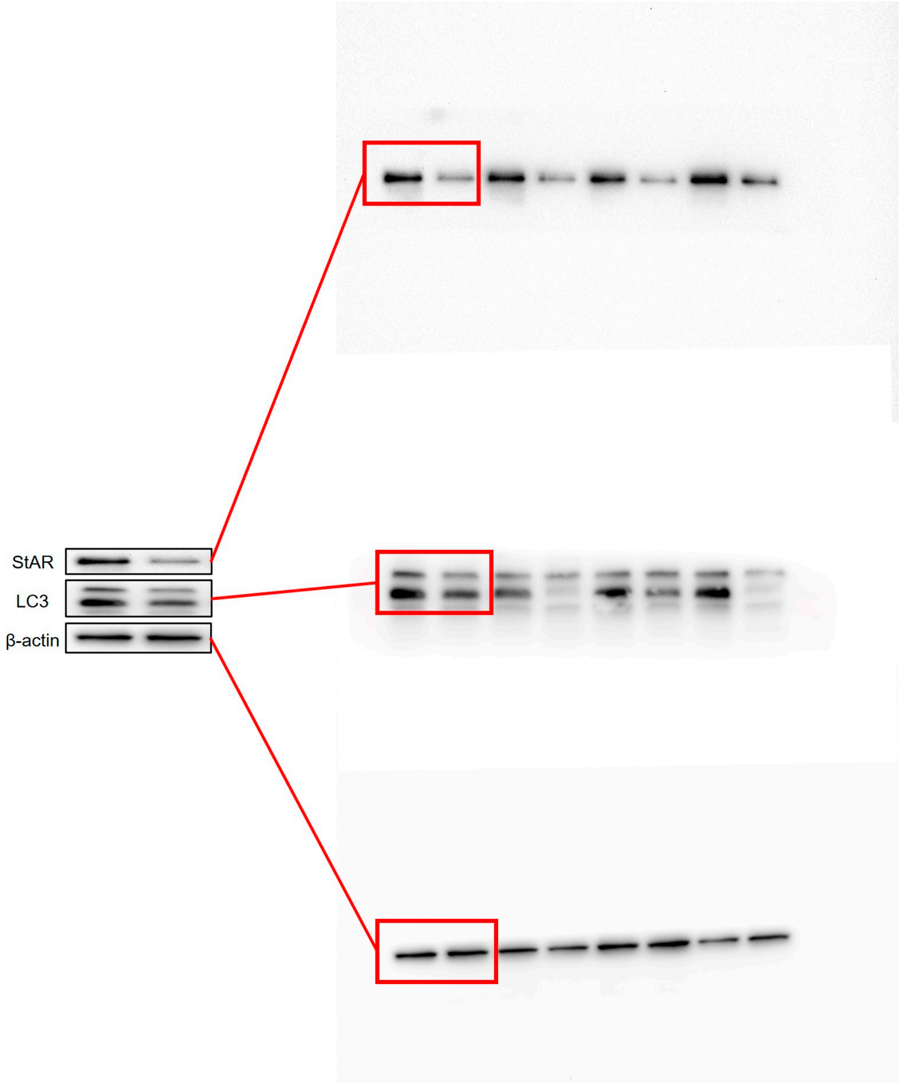

Figure S12. The uncropped counterpart of Figure 6A.

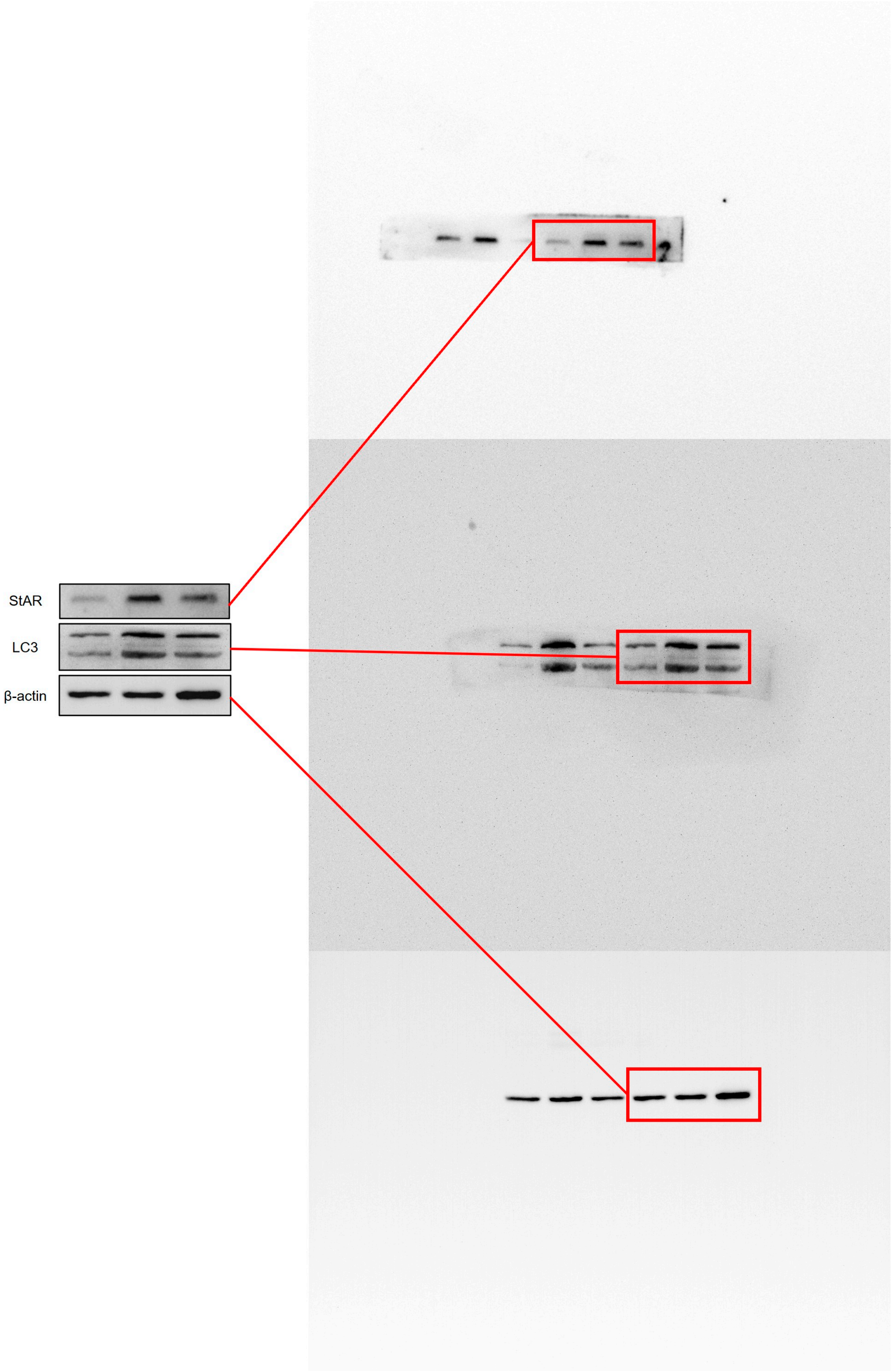

Figure S13. The uncropped counterpart of Figure 6C.

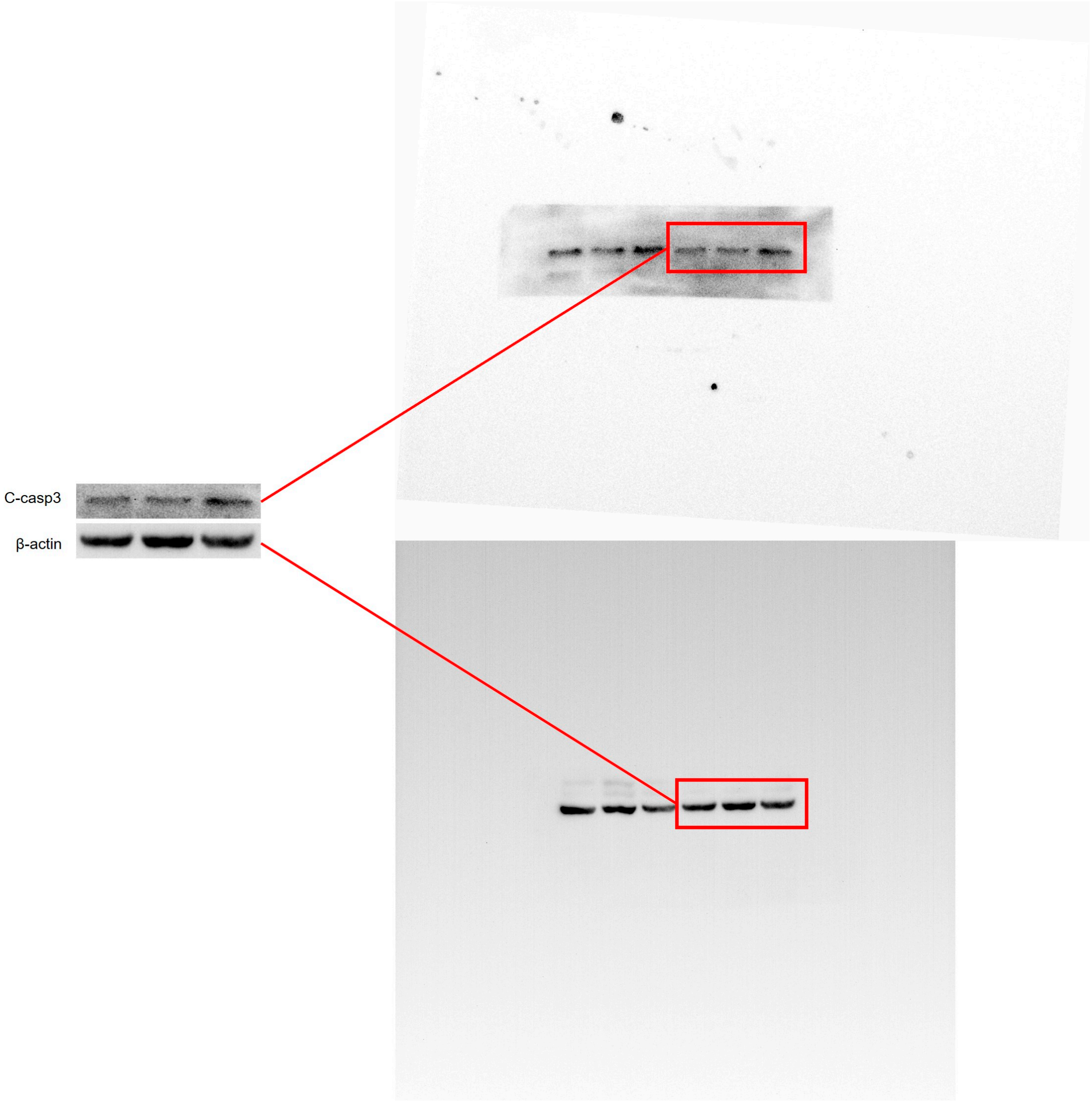

Figure S14. The uncropped counterpart of Figure 6D.

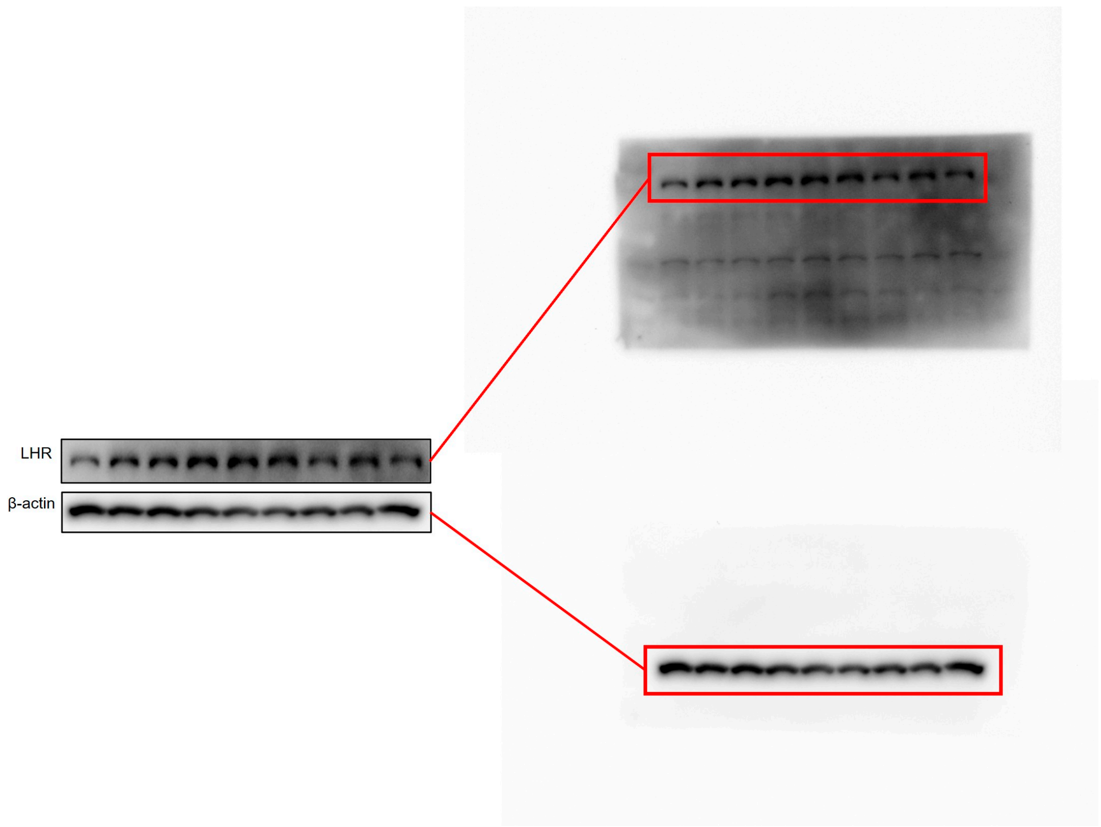

Figure S15. The uncropped counterpart of Figure 6F.

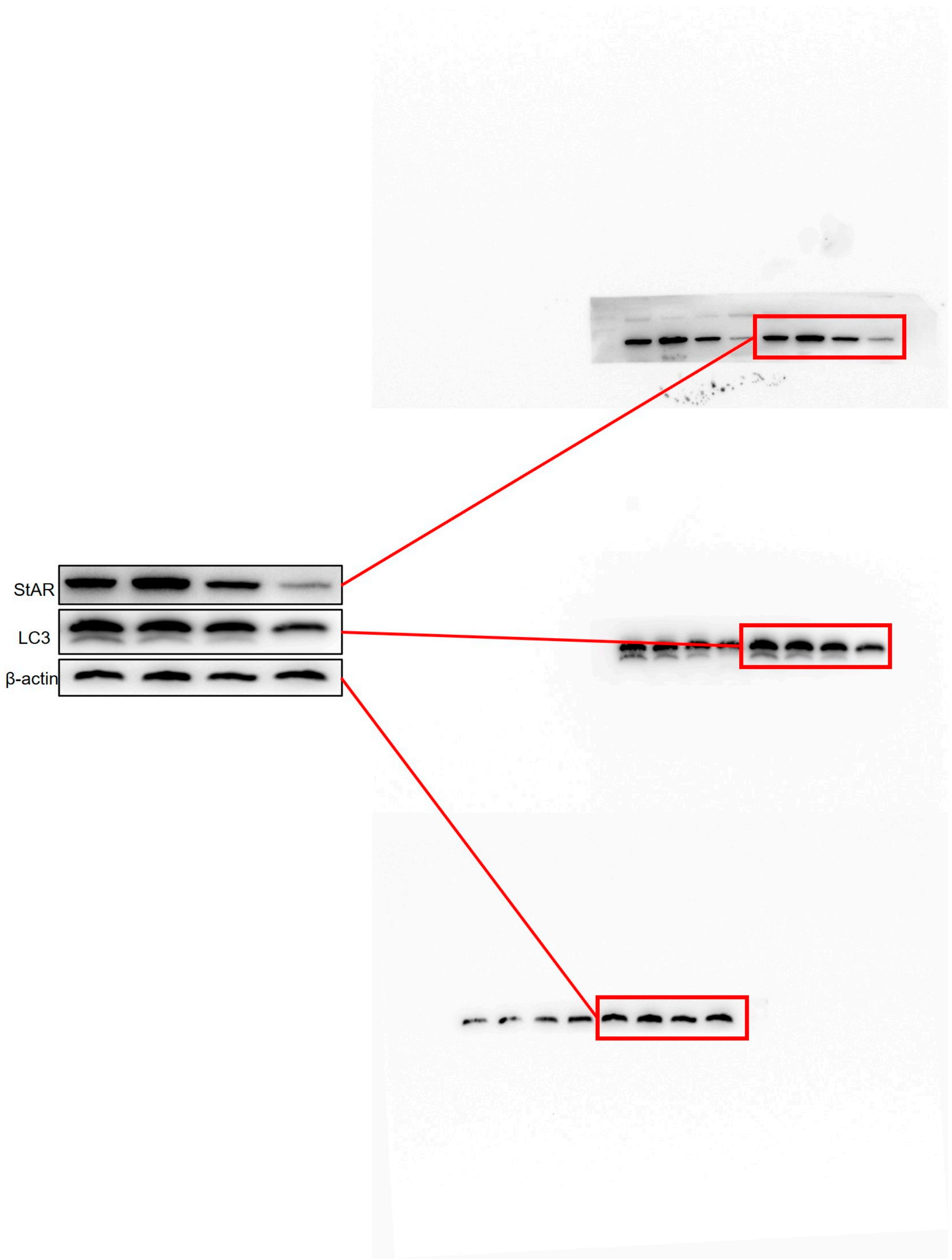

Figure S16. The uncropped counterpart of Figure 7A.

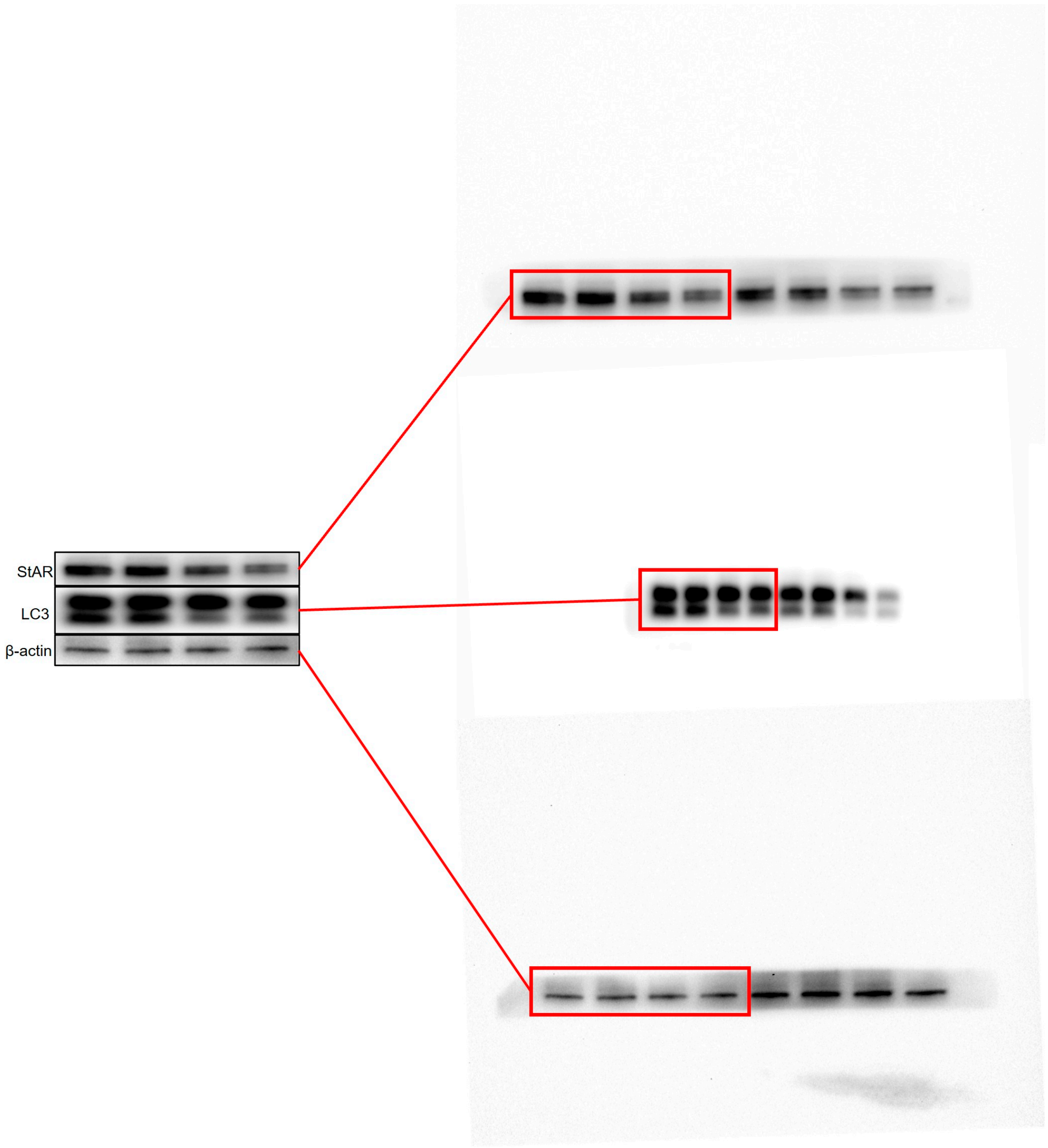

Figure S17. The uncropped counterpart of Figure 7B.

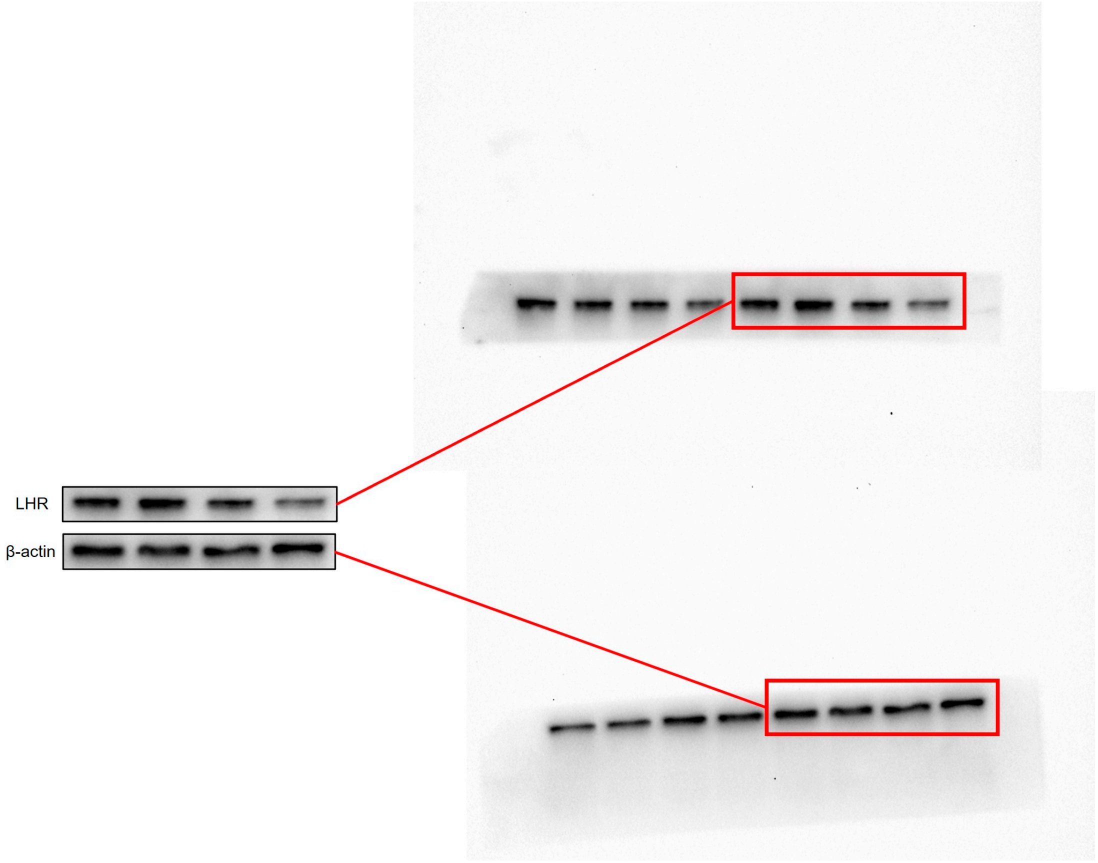

Figure S18. The uncropped counterpart of Figure 7C.

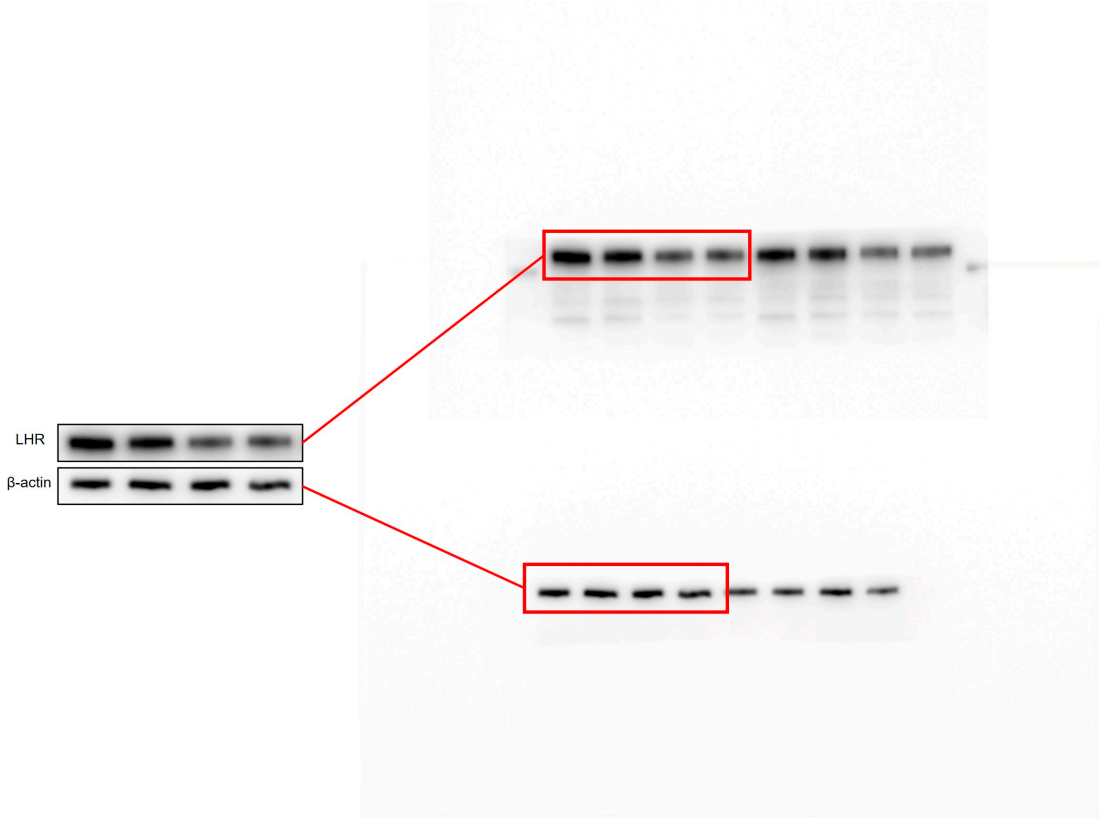

Figure S19. The uncropped counterpart of Figure 7D.

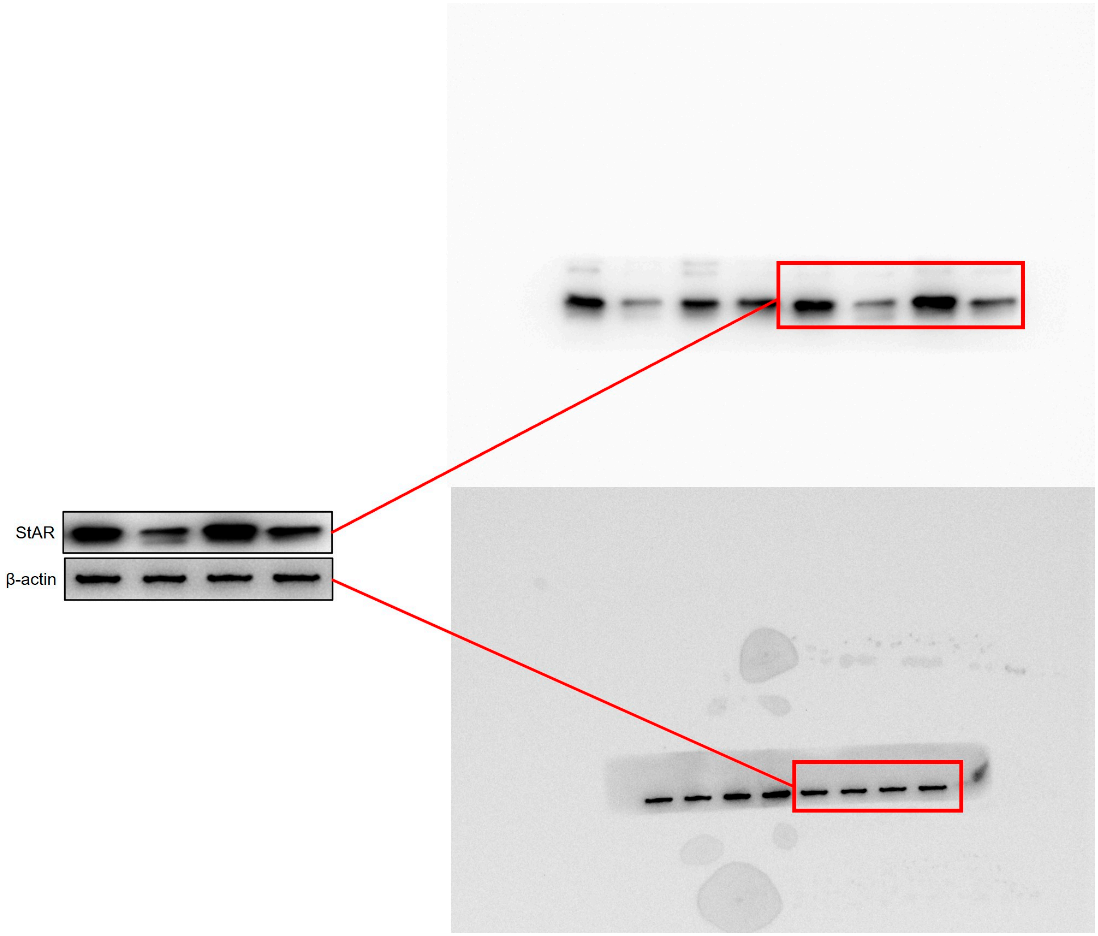

Figure S20. The uncropped counterpart of Figure 7E.

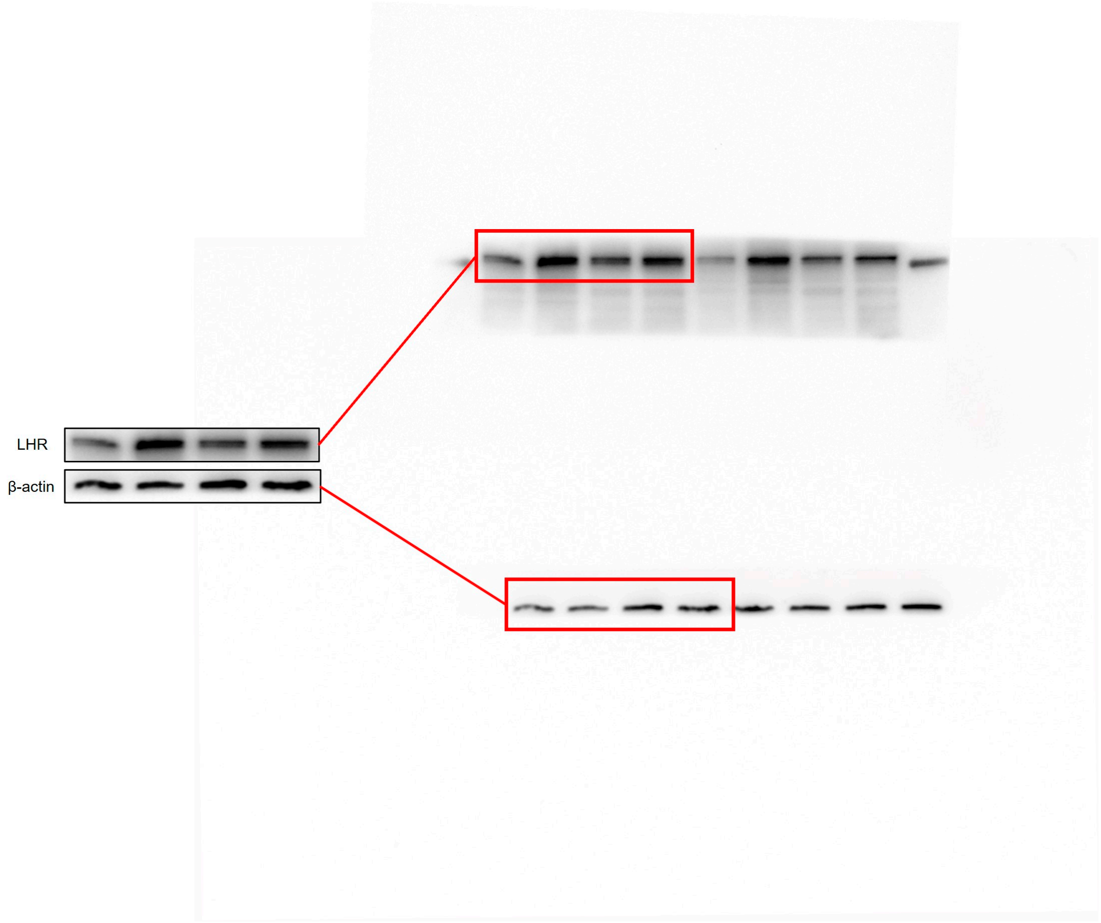

Figure S21. The uncropped counterpart of Figure 7F.

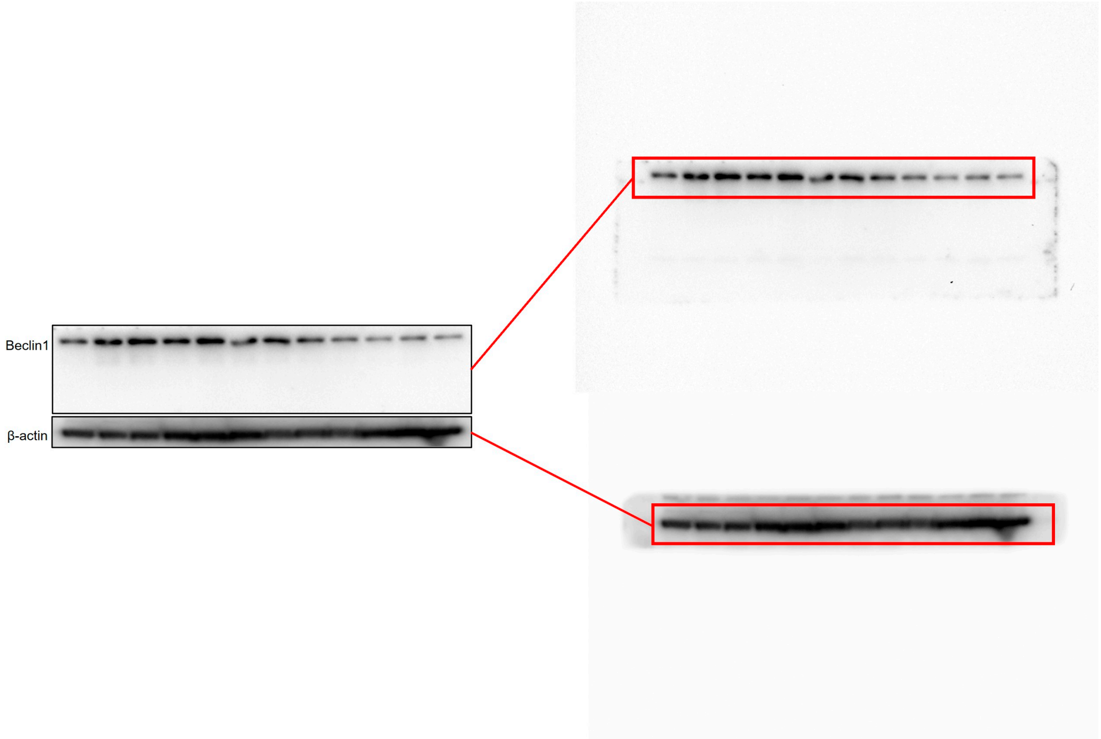

Figure S22. The uncropped counterpart of Figure 7G.

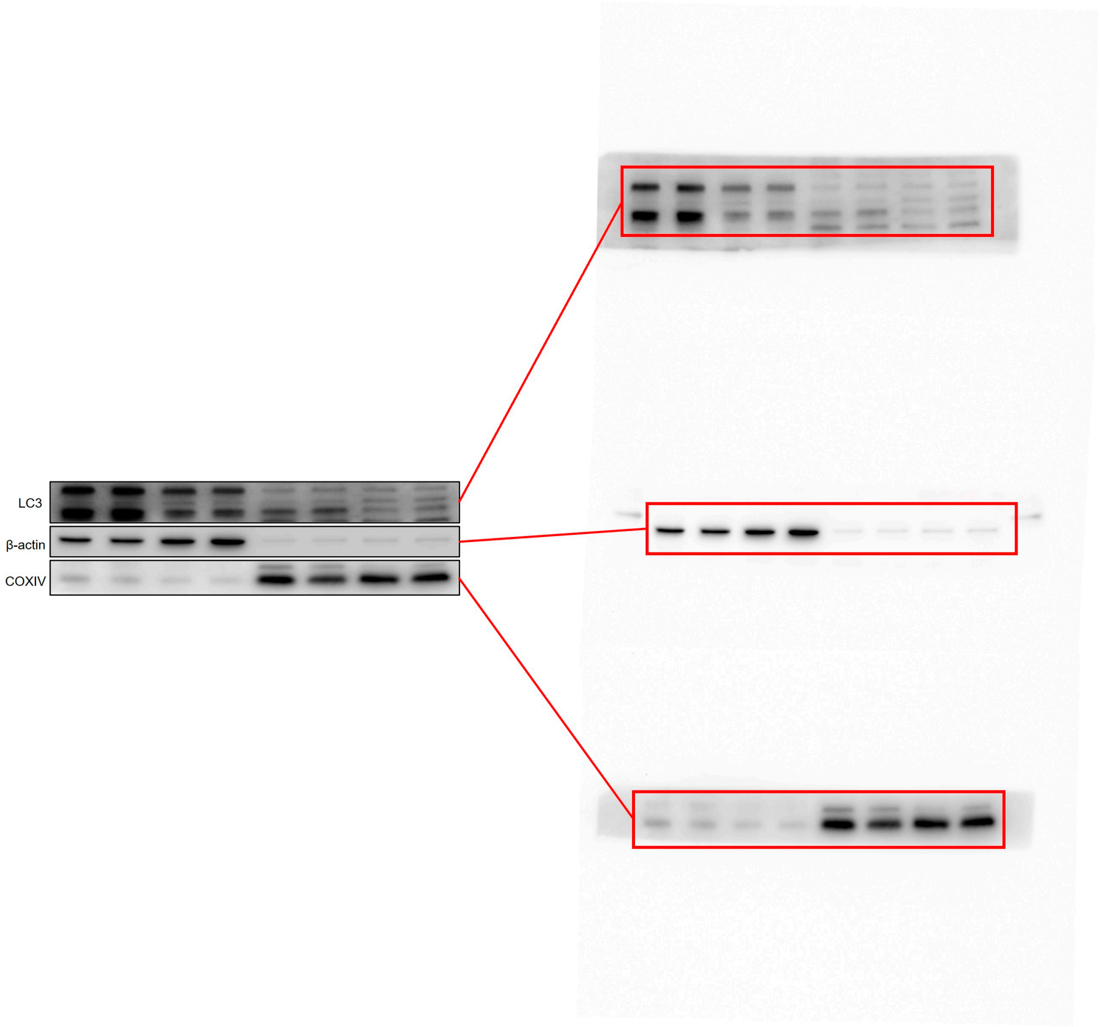

Figure S23. The uncropped counterpart of Figure 7H.

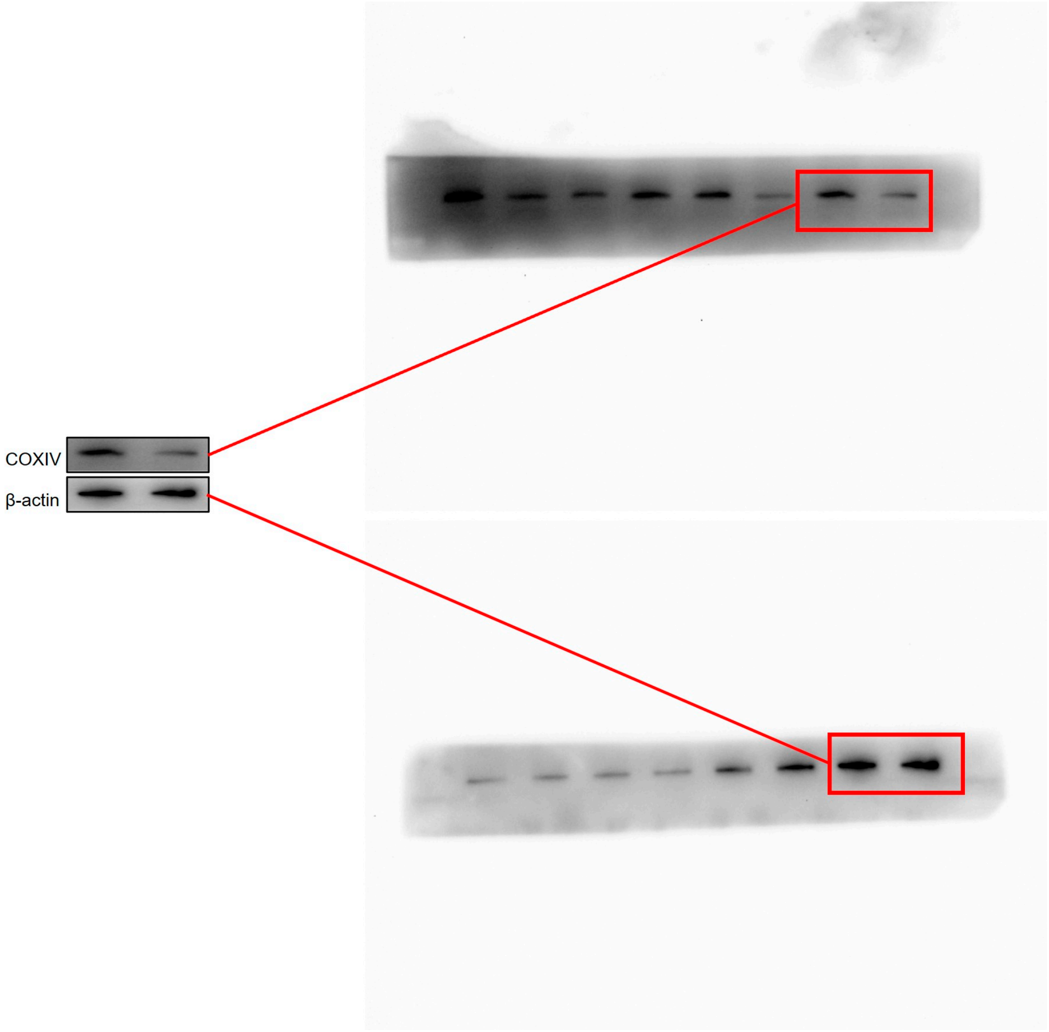

Figure S24. The uncropped counterpart of Figure 8A.

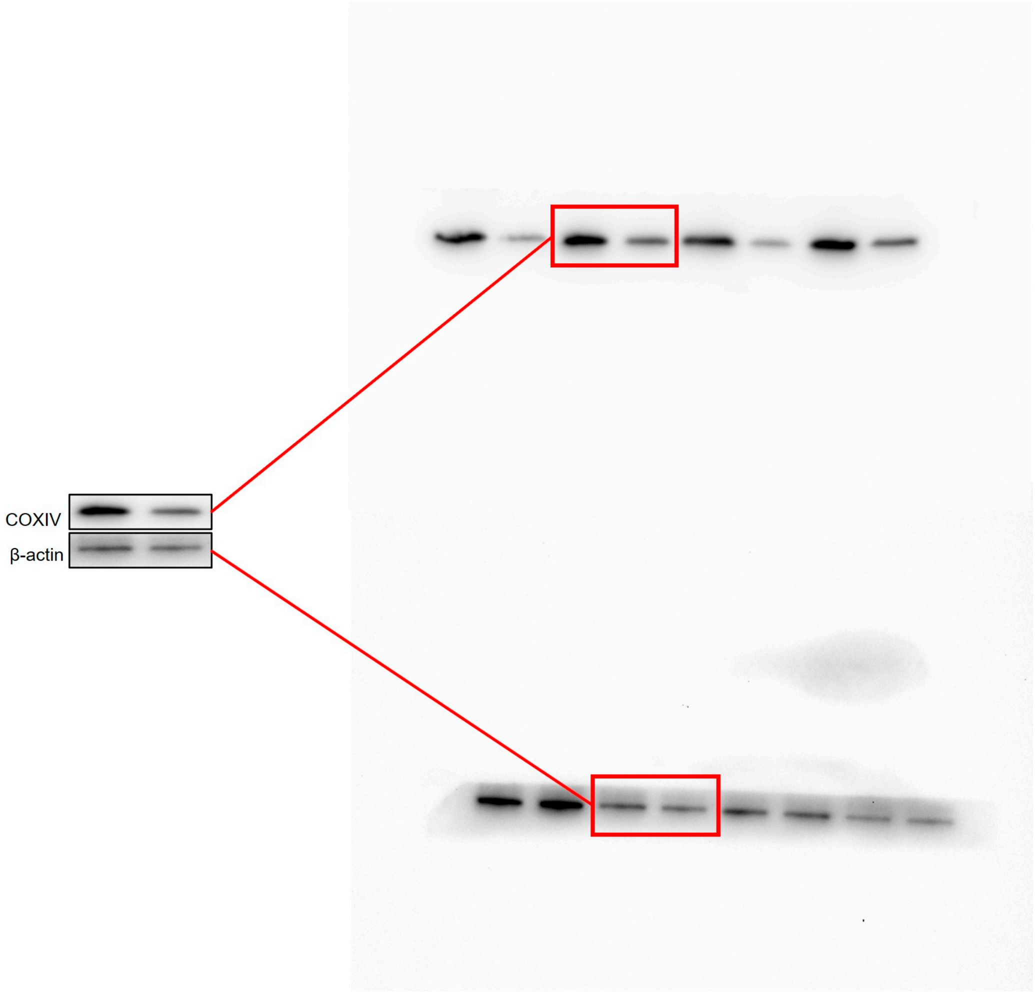

Figure S25. The uncropped counterpart of Figure 8B.

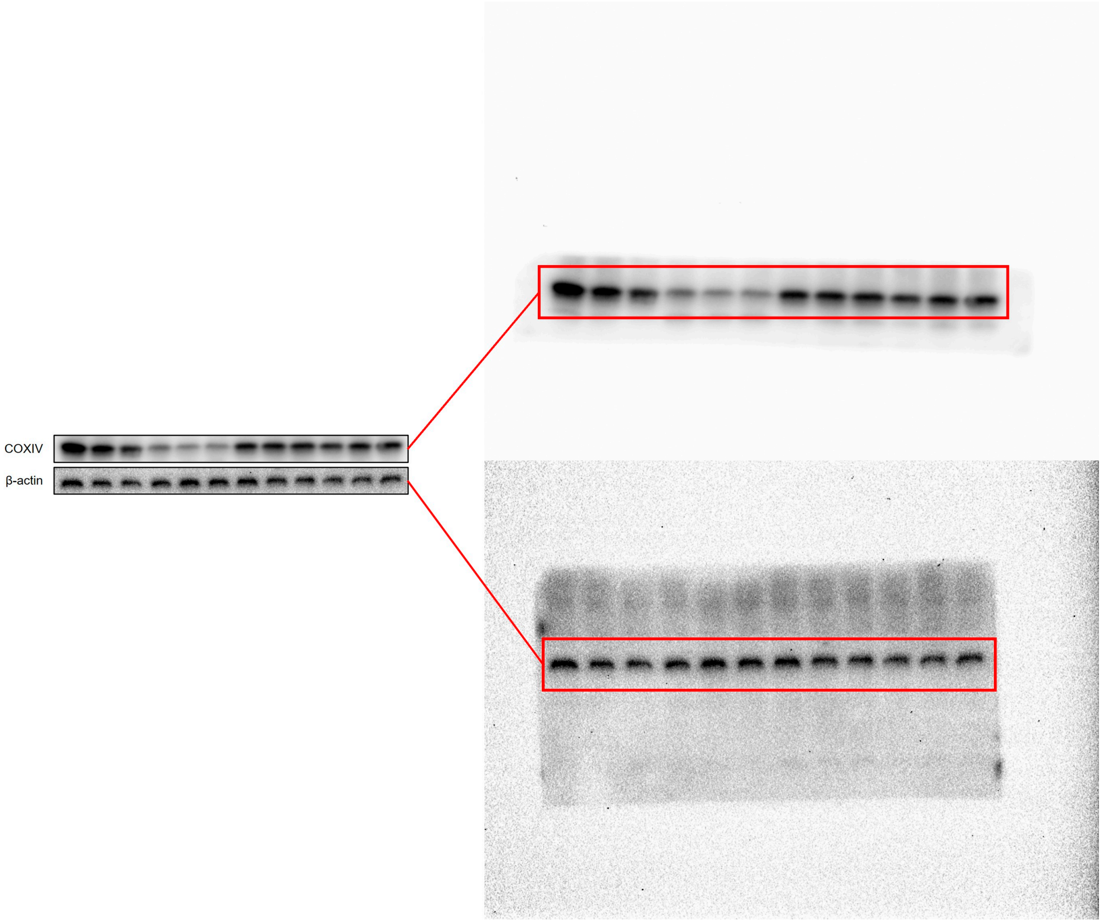

Figure S26. The uncropped counterpart of Figure 8C.

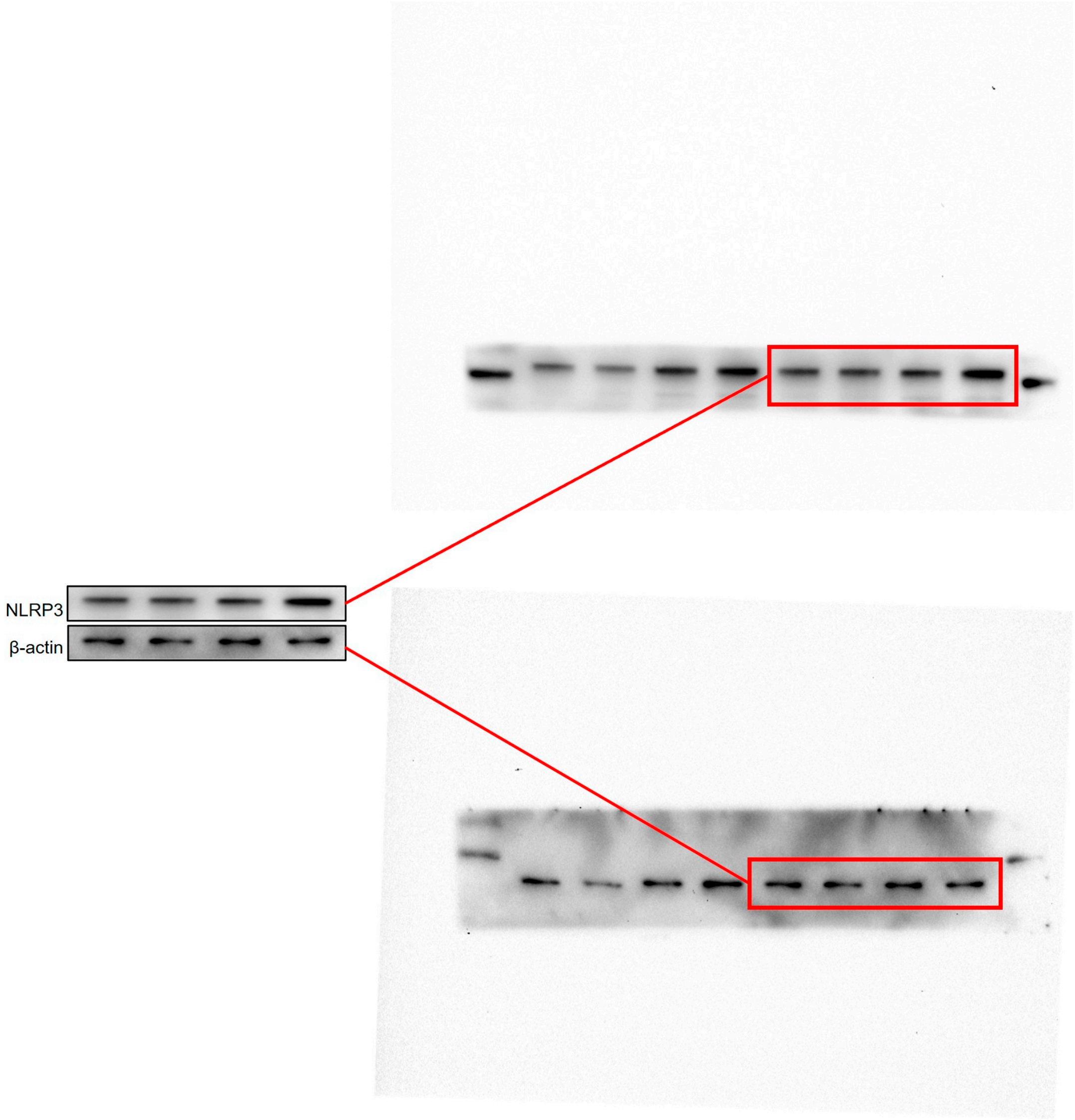

Figure S27. The uncropped counterpart of Figure 9A.

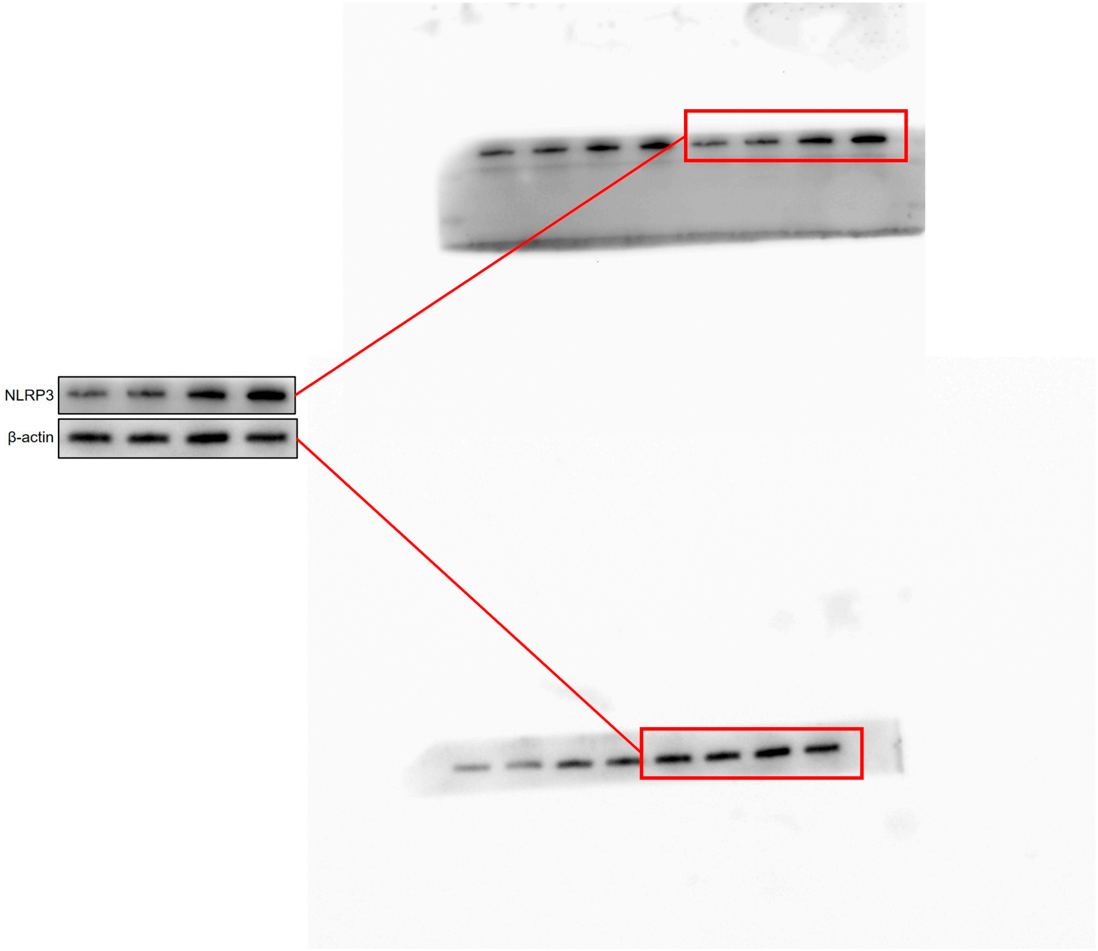

Figure S28. The uncropped counterpart of Figure 9B.

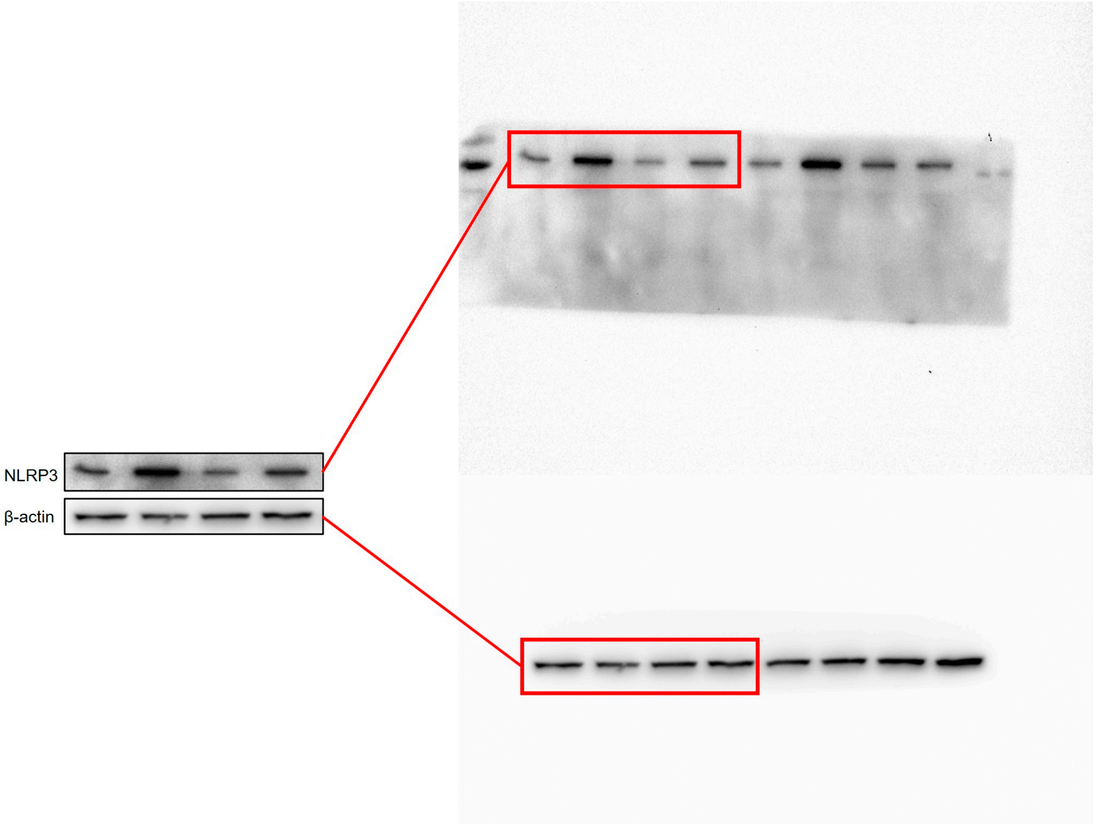

Figure S29. The uncropped counterpart of Figure 9C.

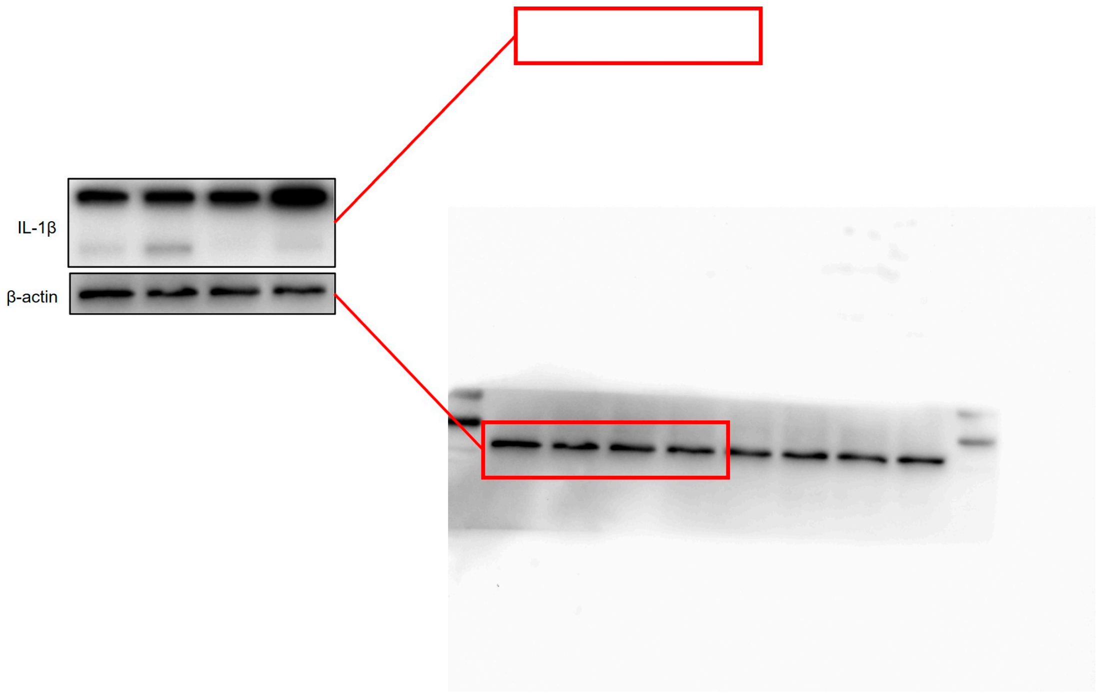

Figure S30. The uncropped counterpart of Figure 9D.

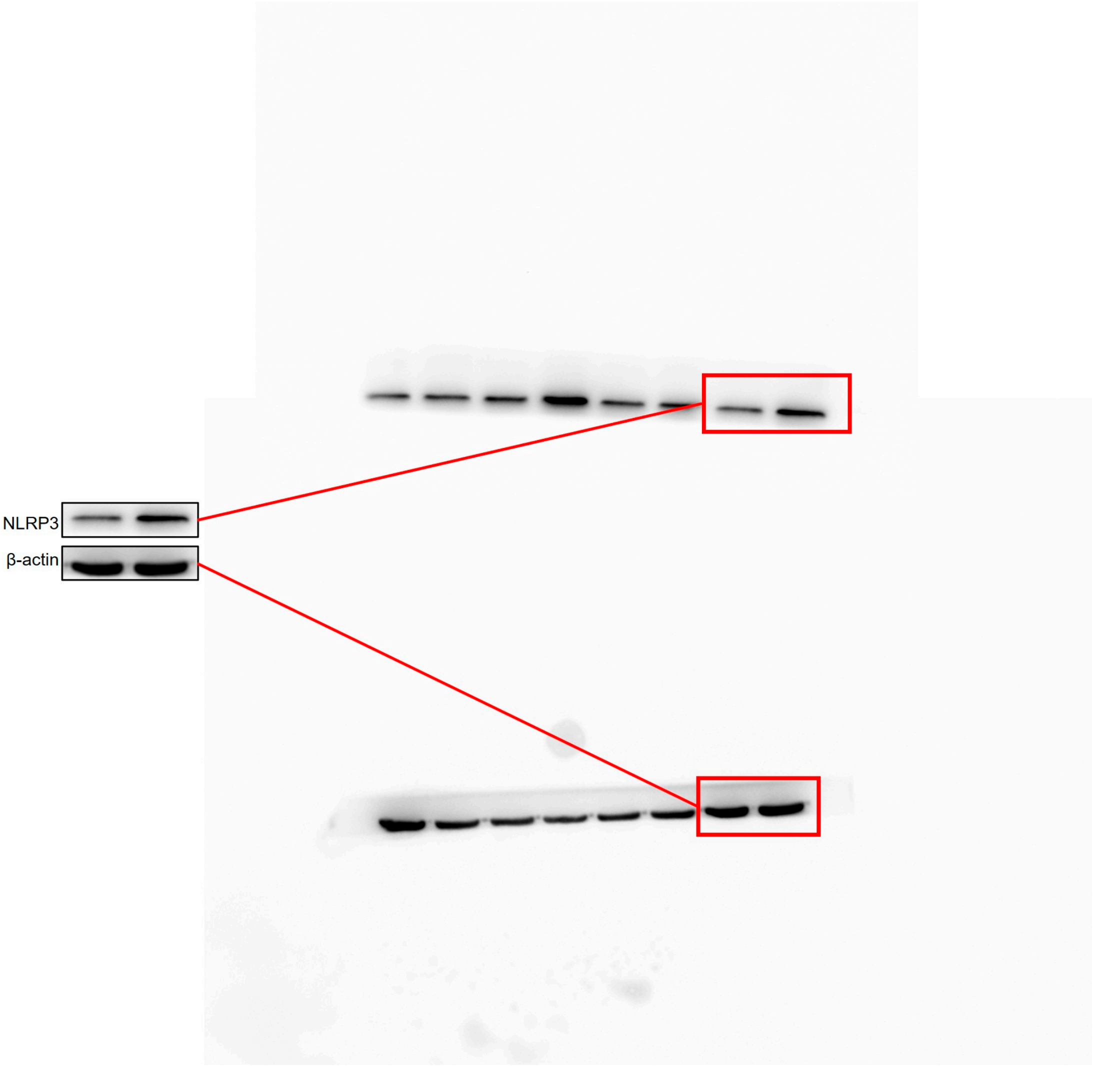

Figure S31. The uncropped counterpart of Figure 9E.

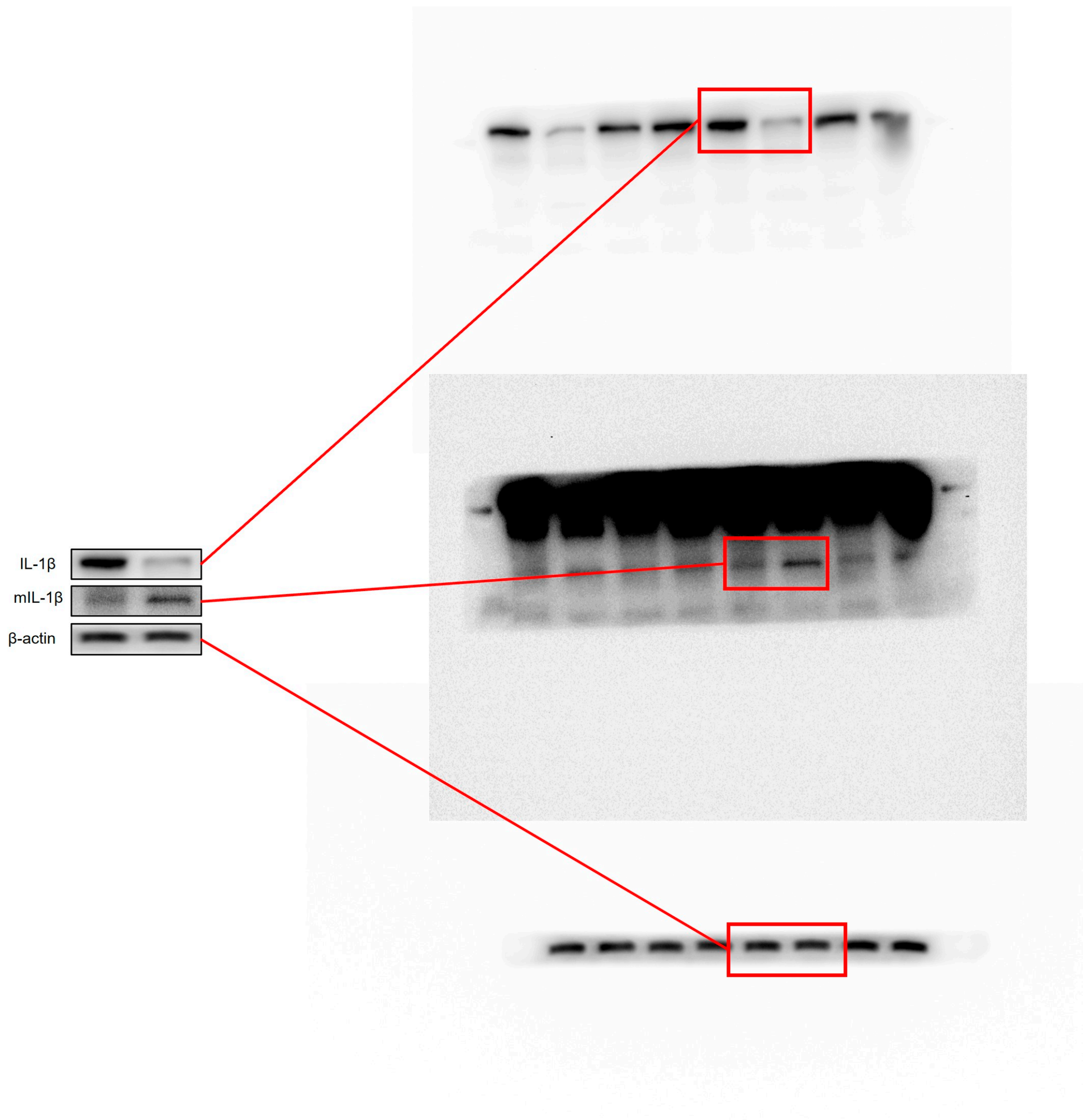

Figure S32. The uncropped counterpart of Figure 9F.

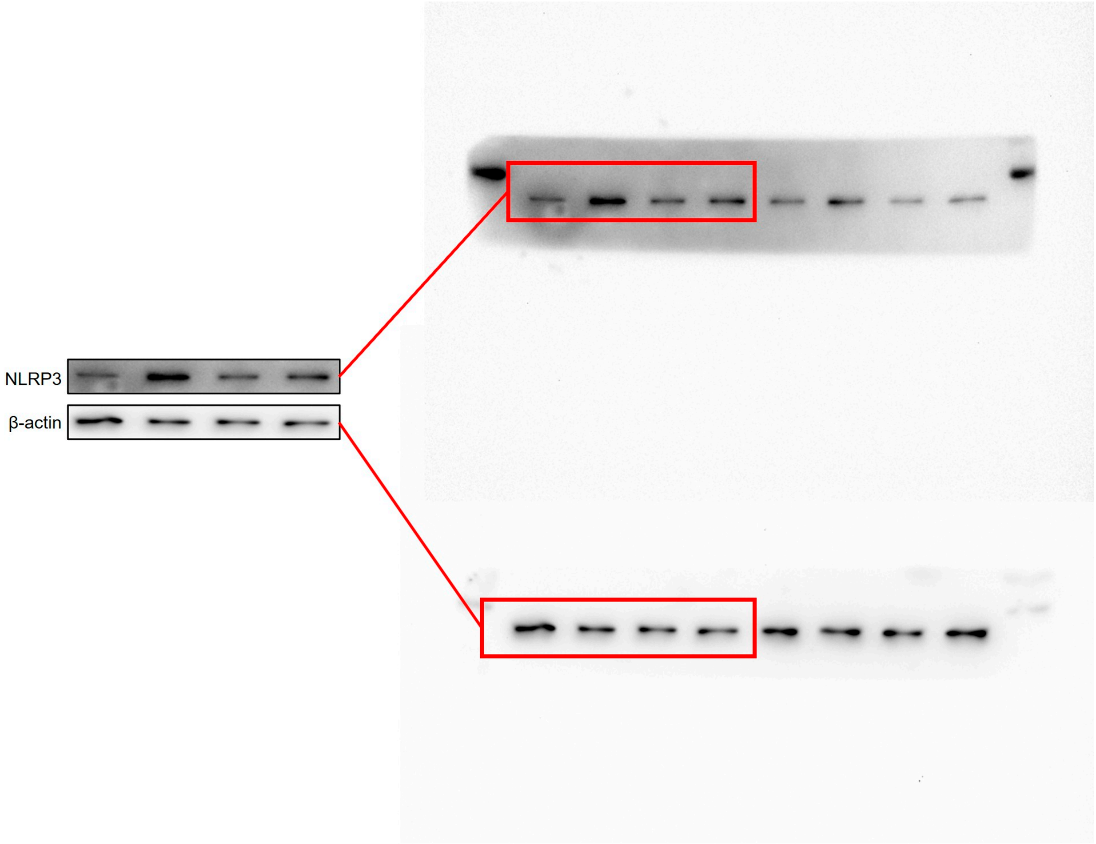

Figure S33. The uncropped counterpart of Figure 10C.

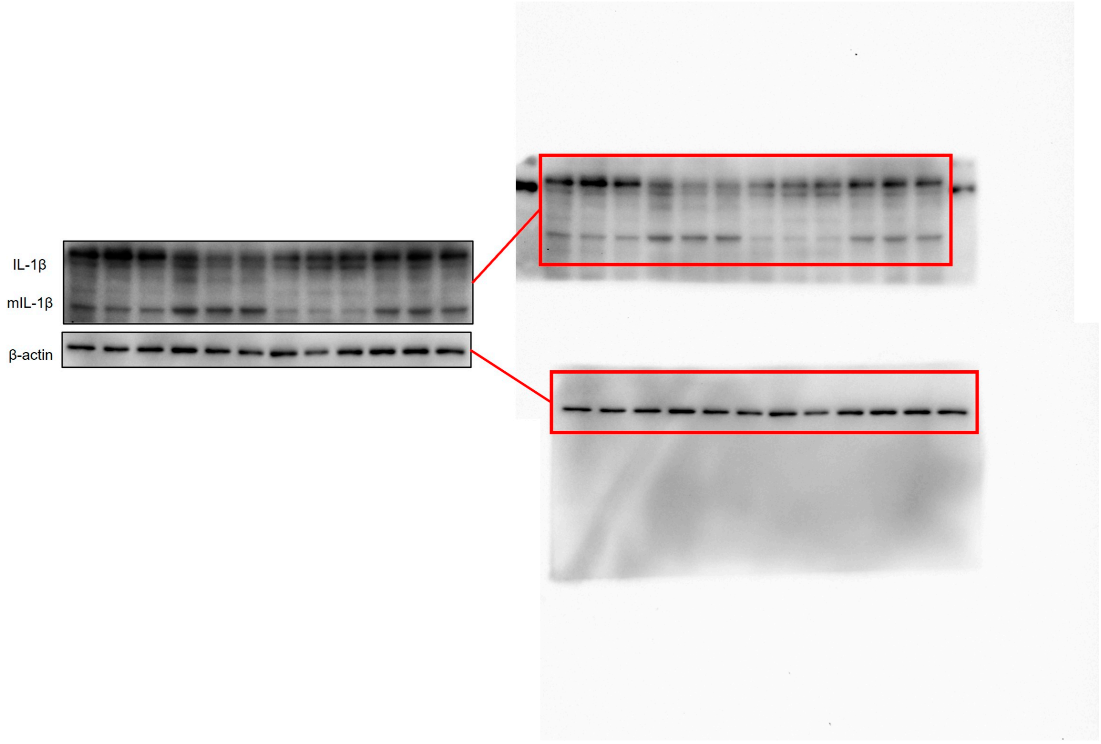

Figure S34. The uncropped counterpart of Figure 10D.

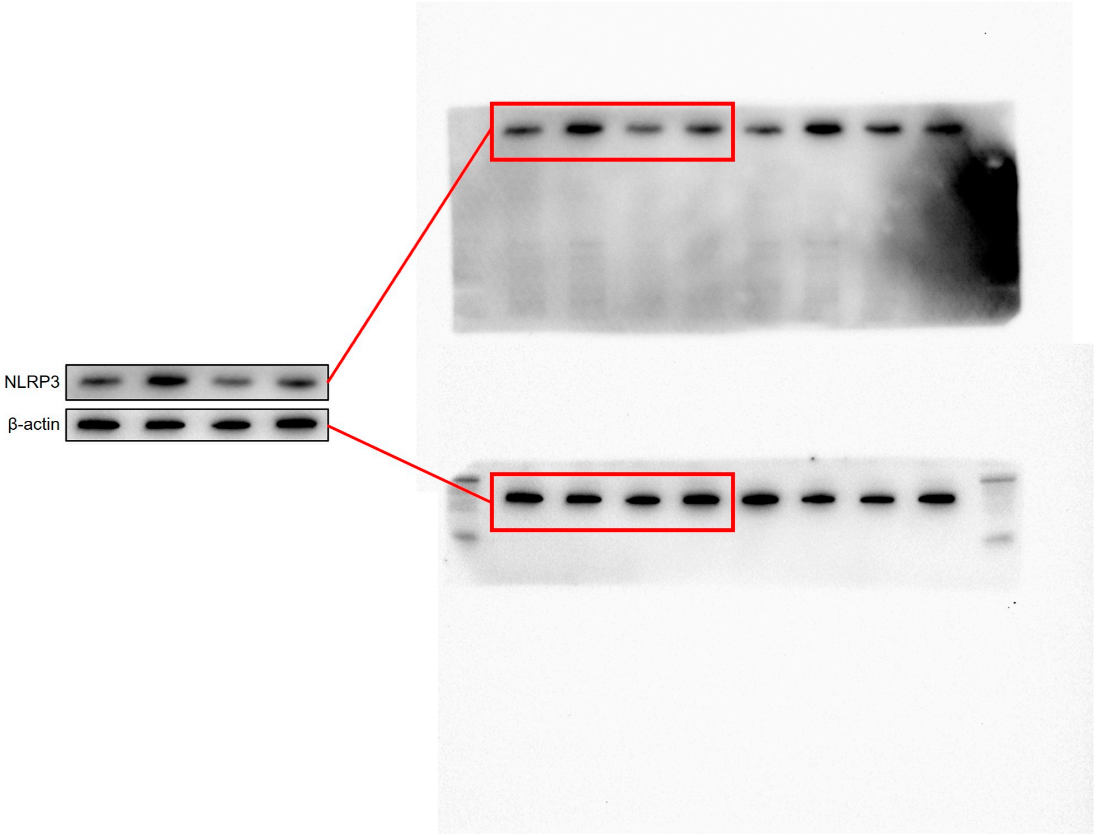

Figure S35. The uncropped counterpart of Figure 10E.

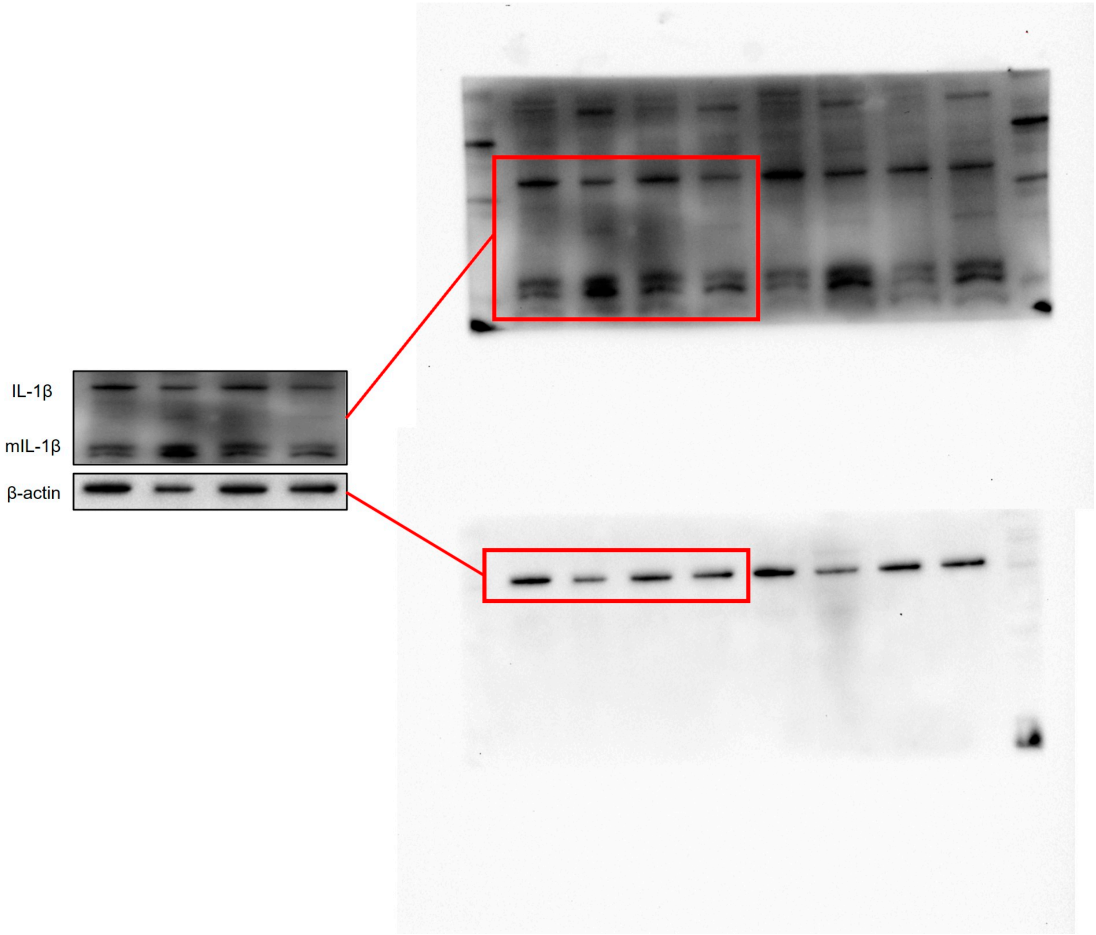

Figure S36. The uncropped counterpart of Figure 10F.

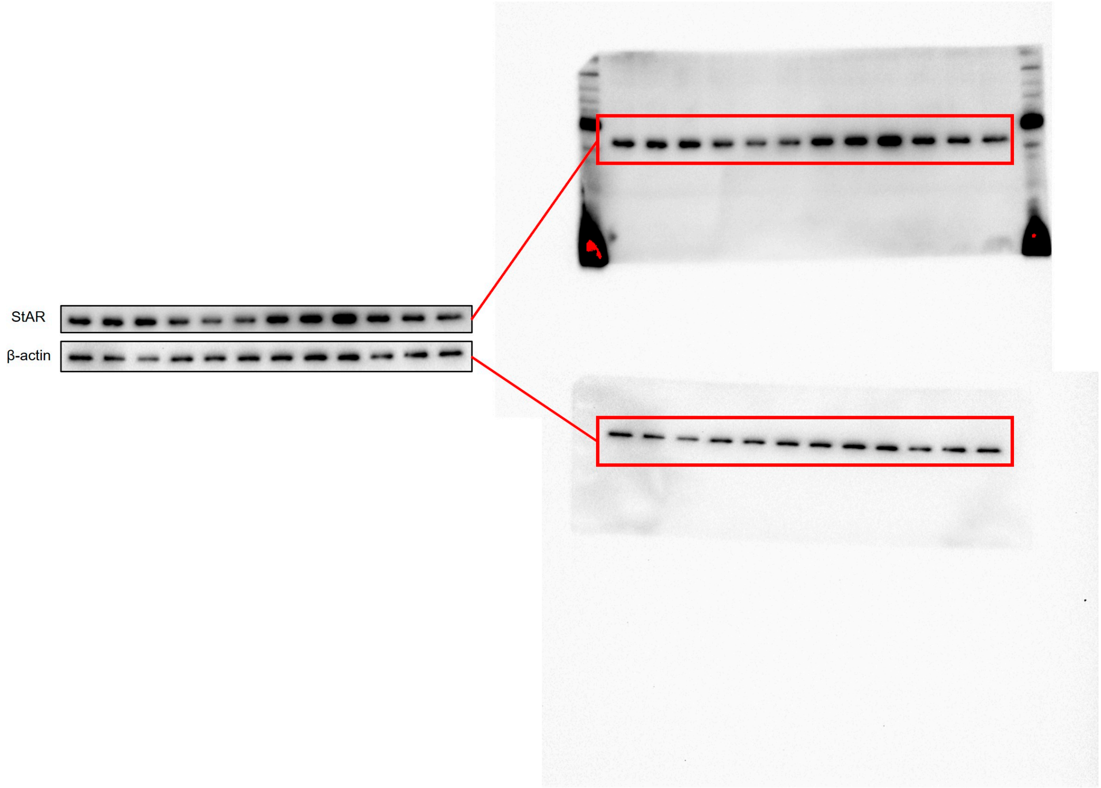

Figure S37. The uncropped counterpart of Figure 10G.

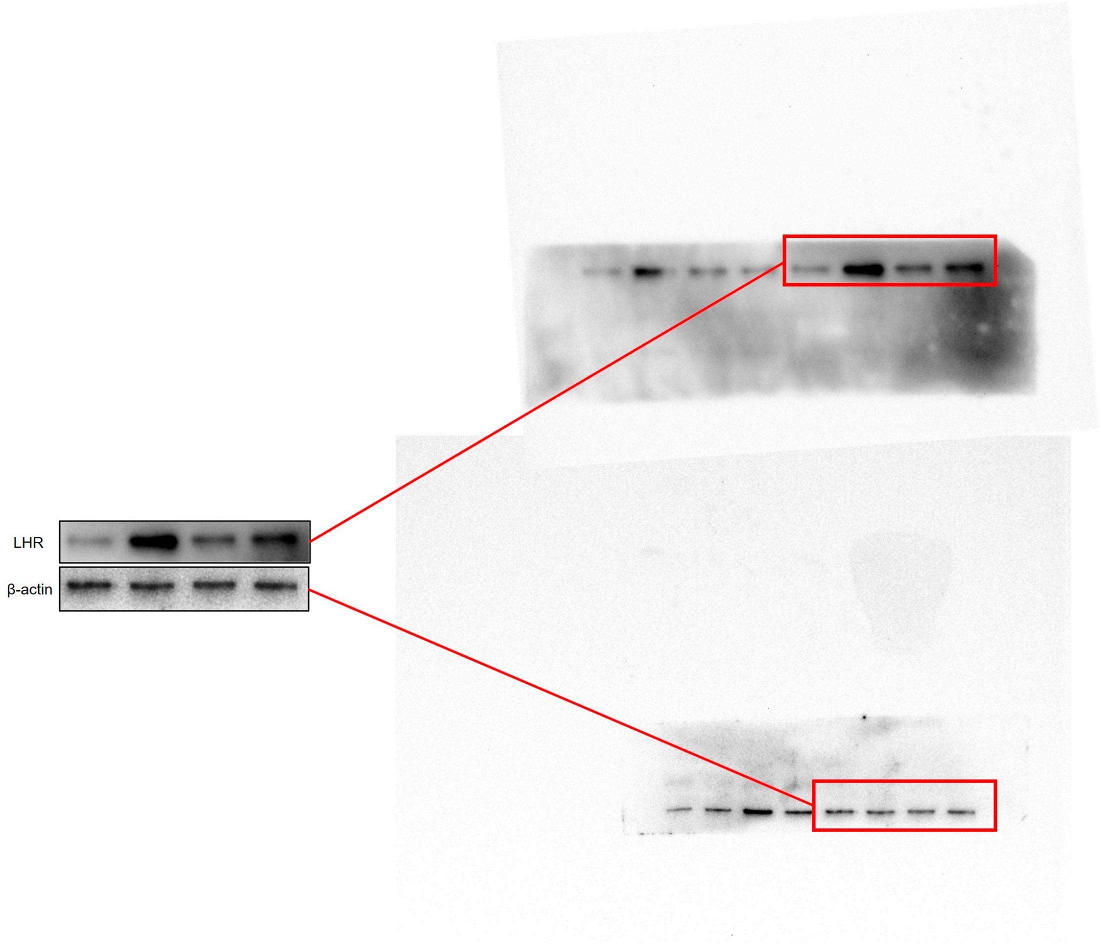

Figure S38. The uncropped counterpart of Figure 10H.

Table S1 Primer sequences for real-time PCR

Primer ID	Forward primer sequence (5'-3')	Reverse primer sequence (5'-3')
Hif-1α	TCA AGT CAG CAA CGT GGA AG	TAT CGA GGC TGT GTC GAC TG
Beclin1	ATG GAG GGG TCT AAG GCG TC	TCC TCT CCT GAG TTA GCC TCT
Bnip3	TCC TGG GTA GAA CTG CAC TTC	GCT GGG CAT CCA ACA GTA TTT
LC3	GGC TAC GGC TAC TAT CGC AC	AGG AGG GCA TGA CAA AGG AGA
Atg5	TGT GCT TCG AGA TGT GTG GTT	GTC AAA TAG CTG ACT CTT GGC AA
Pgk1	ATG TCG CTT TCC AAC AAG CTG	GCT CCA TTG TCC AAG CAG AAT
Ldha	TGT CTC CAG CAA AGA CTA CTG T	GAC TGT ACT TGA CAA TGT TGG GA
Pdk1	GGA CTT CGG GTC AGT GAA TGC	TCC TGA GAA GAT TGT CGG GGA
β-actin	GGC TGT ATT CCC CTC CAT CG	CCA GTT GGT AAC AAT GCC ATG T
